# A Causality Perspective of Genomic Breed Composition for Composite Animals

**DOI:** 10.3389/fgene.2020.546052

**Published:** 2020-10-30

**Authors:** Xiao-Lin Wu, Zhi Li, Yangfan Wang, Jun He, Guilherme J. M. Rosa, Ryan Ferretti, John Genho, Richard G. Tait, Jamie Parham, Tom Schultz, Stewart Bauck

**Affiliations:** ^1^Biostatistics and Bioinformatics, Neogen GeneSeek Operations, Lincoln, NE, United States; ^2^Department of Animal Sciences, University of Wisconsin, Madison, WI, United States; ^3^Ministry of Education Key Laboratory of Marine Genetics and Breeding, Ocean University of China, Qingdao, China; ^4^College of Animal Science and Technology, Hunan Agricultural University, Changsha, China

**Keywords:** beef cattle, crossbred animals, genomic composition, SNP arrays, path analysis

## Abstract

Genomic breed composition (GBC) of an individual animal refers to the partition of its genome according to the inheritance from its ancestors or ancestral breeds. For crossbred or composite animals, knowing their GBC is useful to estimate heterosis, to characterize their actual inheritance from foundation breeds, and to make management decisions for crossbreeding programs. Various statistical approaches have been proposed to estimate GBC in animals, but the interpretations of estimates have varied with these methods. In the present study, we proposed a causality interpretation of GBC based on path analysis. We applied this method to estimating GBC in two composite breeds of beef cattle, namely Brangus and Beefmaster. Three SNP panels were used to estimate GBC: a 10K SNP panel consisting of 10,226 common SNPs across three GeneSeek Genomic Profiler (GGP) bovine SNP arrays (GGP 30K, GGP 40K, and GGP 50K), and two subsets (1K and 5K) of uniformly distributed SNPs. The path analysis decomposed the relationships between the ancestors and the composite animals into direct and indirect path effects, and GBC was measured by the relative ratio of the coefficients of direct (D-GBC) and combined (C-GBC) effects from each ancestral breed to the progeny, respectively. Estimated GBC varied only slightly between different genotyping platforms and between the three SNP panels. In the Brangus cattle, because the two ancestral breeds had a very distant relationship, the estimated D-GBC and C-GBC were comparable to each other in the path analysis, and they corresponded roughly to the estimated GBC from the linear regression and the admixture model. In the Beefmaster, however, the strong relationship in allelic frequencies between Hereford and Shorthorn imposed a challenge for the linear regression and the admixture model to estimated GBC reliably. Instead, D-GBC by the path analysis included only direct ancestral effects, and it was robust to bias due to high genomic correlations between reference (ancestral) breeds.

## Introduction

Genomic breed composition (GBC) of an individual animal refers to the partition of its genome according to the inheritance from its ancestors or ancestral breeds. At the DNA level, every individual has two haplotypes, which are linages of genes and markers. One haplotype is inherited from the father and the other from the mother. In crossbreeding, a haplotype segment is usually present in many individuals as descendants of a common ancestor from which the segment originates. It is also possible that one progeny can carry two segments that are identical-by-status (IBS), meaning identical by chance, because they are not inherited from the same ancestor. The chance of IBS, however, is minimal if many markers are included in the segments, e.g., based on runs of homozygosity (ROH), which are long DNA segments containing consecutive homozygous loci (Ferencakovic et al., 2011; Purfield et al., 2012). The information about GBC is very useful in many aspects. For purebred animals, knowing their genomic composition can help the registry of purebred animals when the pedigree is missing (Kuehn et al., 2014; Norman et al., 2016) or the identification of population structures (Pritchard et al., 2000; Pickrell and Pritchard, 2012). For crossbred or composite animals, GBC is often used to estimate heterozygosity, to understand their breeding history, to characterize their actual inheritance from foundation breeds, and to make management decisions for crossbreeding programs (VanRaden and Cooper, 2015; Akanno et al., 2017; Gobena et al., 2018; He et al., 2018).

Various statistical methods have been proposed to estimate GBC (Pritchard et al., 2000; Tang et al., 2005; Frkonja et al., 2012; Bansal and Libiger, 2015), but the interpretations of estimates have varied across methods. For example, linear regression estimated the GBC of an individual by adjusted regression coefficients of coded genotypes of each animal as the progeny on the ancestral allele frequencies (Chiang et al., 2010; Kuehn et al., 2014; VanRaden and Cooper, 2015). The regression coefficients, however, have no precise interpretation of GBC because they can be any real values, not bounded between 0 and 1. Statistically speaking, linear regression is more of a prediction method rather than an appropriate approach for quantifying genomic causality relationships. When applying the least squares, for example, the linear regression equation is fitted by minimizing the discrepancy between the observed dependent values and their fitted value given by the linear equation. Hence, the usefulness of such an equation is that it gives the best or closest prediction, independently of the meaning of predictors, and it provides no exact indication on the causality relationships of these variables. Likewise, estimated GBC using a genomic prediction model is also based on estimated variable effects, which is more of a prediction by its nature than of causality (Akanno et al., 2017). Besides, a multiple regression model is not robust to high correlations between independent variables. In reality, however, modern cattle breeds are genetically related to various extent (Ajmone-Marsan et al., 2010). Such strong relationships between breeds give rise to the problem of multicollinearity, which in turn leads to ill-estimated linear regression coefficients, e.g., when obtained with least-squares. Another approach for estimating GBC is the admixture model, which postulates that an observed genotype is an instance of a multinomial distribution with the genotype probability being a mixture of those of their ancestors. In this case, the GBC of an individual animal is estimated by the weights of the admixture (Bansal and Libiger, 2015). Like in the case of the linear regression approach, if ancestors are highly correlated, it also imposes a challenge to precisely estimate the weights for the admixture model.

Path analysis has been developed to model causal relationships between variables. In the path analysis, exogenous (independent) variables produce both direct and indirect path effects on one or more endogenous (dependent) variables. The indirect path effects due to the correlations between the exogenous variables are also referred to as the correlational effects (Land, 1969). Path analysis was initially developed by Sewall Wright in a series of general essays (Wright, 1921, 1934, 1954, 1960a,b) as an analytical tool for quantitative genetics to measure “the direct influence along each separate path in such a system and to find the degree to which variation of a given effect is determined by each particular cause” (Wright, 1921).

In the present study, we proposed the use of path analysis to decompose the causality relationships between composite (or crossbred) animals and their putative ancestors (or reference breeds) and to estimate GBC of individual animals in terms of the relative determination of respective ancestral (or reference) breeds. Two measures of GBC were used, one accounting only for the direct path effects of each reference breed, and the other including both direct and indirect path effects for each reference breed. The indirect path effects were attributable to the correlations between the reference breeds. Estimated GBC from the path analysis was compared with those obtained using the linear regression and the admixture model, and their similarities and dissimilarities were discussed as well.

## Materials and Methods

### Animals, Genotypes, and SNP Panels

The genotypes of 150,676 animals sampled from two composite breeds and eight reference breeds of beef cattle were used in the present study (Table 1). The composites included 7,605 Beefmaster and 7,969 Brangus. The reference animals included 45,396 Angus, 2,320 Brahman, 10,423 Hereford, 1,587 Shorthorn, 17,769 Gelvieh, 7,680 Limousin, 23,722 Simmental, and 26,689 Wagyu before data cleaning. These animals were genotyped on GeneSeek Genomic Profiler (GGP) LD V3 (GGP 30K) bovine SNP chip (32,179 SNPs), GGP bovine SNP 40K chip (40,660 SNPs), and GGP bovine 50K bovine SNP chip (49,463 SNPs), respectively (Neogen GeneSeek Operations, Lincoln, NE). The GGP 40K bovine SNP chip included common 31,901 SNPs with the GGP 30K. The GGP bovine 50K had 11,333 SNPs in common with GGP bovine 30K SNP chip and 16,369 SNPs in common with GGP bovine 50K SNP chip. Data cleaning removed monomorphic SNPs across all breeds, and SNPs with 10% missing in each breed. After data cleaning, 10,226 common SNPs (referred to as the 10K SNP panel) across the three GGP bovine SNP chips were retained. Then, from the 10K set, two sets: (1) 1,000 uniformly distributed SNPs (1K panel), and (2) 5,000 uniformly distributed SNPs (5K panel), were selected using the selectSNP package (Wu et al., 2016). A map view of the three SNP panels is shown in Supplementary Figure 1. These three SNP panels were used to estimated GBC for the composite animals.

**TABLE 1:** Number of genotyped animals and number of SNPs on GeneSeek Genomic Profiler (GGP) 30K (GGP 30K), 40K (GGP 40K), and 50K (GGP 50K) SNP chips used in the present study^a,b^.

Type	Breed	GGP30K		GGP40K		GGP50K		nAnim	
		nAnim	nSNP	nAnim	nSNP	nAnim	nSNP	Before DC	After DC
Composite	Beefmaster	23	32,179	300	40,663	7,282	49,463	7,605	7,605
	Brangus	1,319	32,179	3,053	40,660	3,605	49,463	7,969	7,969
Ancestral	Angus	6,839	32,179	18,198	40,660	20,359	49,463	45,396	45,367
	Brahman	–	–	1,811	30,720	509	43,984	2,320	2,271
	Hereford	4,000	32,179	4,000	40,660	2,423	49,463	10,423	10,414
	Shorthorn	–	–	355	40,660	1,232	49,463	1,587	1,577
Non-ancestral	Gelbvieh	2,763	32,179	5,498	40,660	9,508	49,463	17,769	17,735
	Limousin	373	32,179	2,264	40,660	5,043	46,915	7,680	7,677
	Simmental	3,130	32,179	5,838	40,660	14,754	49,463	23,722	23,697
	Wagyu	1,463	32,179	1,506	40,660	23,720	49,463	26,689	26,364
Sum		19,910		42,823		88,435		152,160	150,676

**^*a*^nAnim = number of genotyped animals; nSNP = Number of SNPs on the chip; ^*b*^nAnim (Before DC vs. After DC) = Total number of animals BEFORE or AFTER data cleaning.**

Data cleaning on reference animals was conducted following He et al. (2018). Briefly, the likelihood that an animal belonged to a specific breed was computed based on a Bayesian multinomial model, assuming independence between SNP loci. Then, outliers with the negative two times the likelihood being greater than two were excluded in each reference population. After data cleaning, 135,102 reference animals from eight breeds remained as the reference animals. Of the eight reference cattle breeds, Brahman is the only *Bos taurus indicus* breed, and it had the most remote relationships with the seven *Bos taurus taurus* cattle breeds. The relationships between the eight reference breeds were depicted by a hierarchical clustering analysis (Murtagh and Legendre, 2014) using the 5K SNP panel and shown in Supplementary Figure 2. All composite animals were included in the subsequent analyses because they were test animals and not used as the reference. Histograms of allele frequencies for the 10K SNPs for the eight reference breeds and the two composite breeds are shown in Supplementary Figure 3. The distributions of allele A frequencies for these breeds (except Brahman) were approximately “bell-shaped,” but they were not typical of a normal distribution. They mostly had “thick” tails, representing SNPs with small minor allele frequencies (MAF). In particular, the distribution of allele A frequencies for Brahman had “outstanding” proportions of SNPs with MAF. These GGP bovine SNP chips (30K, 40K, and 50K) were primarily designed for *Bos* Taurus cattle, not for *Bos* indicus cattle. Possibly, many SNPs could have small MAF or even be monomorphic. It is also possible that there existed population mixture or stratification with this Brahman dataset.

The genomic breed composition (GBC) was estimated in the two composite breeds. Brangus was developed to combine the desirable traits of Angus and Brahman cattle (Briggs and Briggs, 1980). Angus cattle are known for their superior carcass qualities. Moreover, Angus cows are well known for their excellent fertility and their capability for milking. The Brahman has gone through rigorous natural selection and has developed disease resistance, and overall they have hardiness and outstanding maternal instincts. For official registration, a Brangus animal needs to be genetically stabilized at 3/8 Brahman and 5/8 Angus by pedigree, be solid black or red, and polled, and both sire and dam must be recorded with the International Brangus Breeders Association (IBBA) (San Antonio, TX). The Beefmaster was developed in the early 1930s from a crossing of Hereford cows and Shorthorn cows with Brahman bulls (Briggs and Briggs, 1980). The original intention was to produce cattle that could produce economically in the challenging environment of South Texas. Nowadays, these cattle are regarded as a versatile, multipurpose breed because they can be used for both milk and beef production. The exact mixture of the foundation cattle is unknown but is generally thought to be about 25% Hereford, 25% Milking Shorthorn, and 50% Brahman.

### Statistical Methods

#### Linear Regression and the Likelihood-Based Admixture Model

These two models served as the benchmark for comparison in the present study. In the linear regression approach, the genotypes of a crossbred animal are coded to be the proportion (or frequency) of say allele A in the genotype for all involving SNPs across the genome. Then, the coded genotypes are regressed to the corresponding allele A frequencies of SNPs for a set of reference populations (Chiang et al., 2010; Hulsegge et al., 2013; Kuehn et al., 2014). Let AA = 1, AB = 0.5, and BB = 0, which can also be interpreted to be the allele A frequencies at the individual level. Denote *y*_*i*_ to be a *M*×1 vector of genotypes pertaining to animal *i*, where *M* is the number of SNPs involved, and denote *x*_*j*_ to be an *M*×1 vector of allele A frequencies of the *M* SNPs genotype in reference population or breed *j*, for *j* = 1,…,*K* where *K* is the number of breeds. Then, the GBC is estimated based on the following linear model:

(1) $$y_{i}=1\,\mu +\sum_{j=1}^{K}b_{j}\,x_{j}+e_{i}$$

where μ is an intercept, and *b*_*j*_ is the regression coefficient pertaining to population or breed *j*, and *e*_*i*_ is a vector of residuals. Because regression coefficients are not bounded between 0 and 1 by nature, some adjustments are necessary to restrict the sum of the regression coefficients for each animal to be 1 (VanRaden and Cooper, 2015; He et al., 2018).

For crossbred animals whose ancestors originated in different populations, their genetic composition exhibits multiple ancestries associated with multiple different genetic clusters or populations, which therefore can be described by the admixture model (Pritchard et al., 2000; Tang et al., 2005; Alexander et al., 2009; He et al., 2018). The admixture model estimates GBC as the weights for an underlying admixture distribution, which governs the realization of genotypes for individual animals, and each component in the admixture corresponds to the allele frequency of each reference breed. Consider *M* SNPs, each with two alleles A and B. Let there be *T* reference or putatively ancestral populations with allelic frequencies of these SNPs assumedly to be known. Denote *x*_*ij*_ to be the allele frequency of the allele A at the ith SNP in the *j*th population. Following Bansal and Libiger (2015), we estimated the allelic frequencies of SNPs *a priori* and then treated them as known in the admixture model. Let *w*_*j*_ represent the admixture proportion for the *j*th population and *W* = [*w*_1_, *w*_2_,…,*w*_*k*_]′ be the vector of admixture coefficients. Then, weighted allele frequency at SNP *i* given the allele frequencies and the admixture proportions was calculated to be $f_{i}=\sum_{j=1}^{k}x_{i\,j}\,w_{j}$, where *x*_*ij*_ was the allele *A* frequency of the *i*th SNP in the *j*th reference breed. Assuming Hardy-Weinberg equilibrium (HWE) at each SNP locus, the probability of observing genotype *y*_*i*_ at locus *i* is:

(2) $$P\,r\,\left(y_{i}|f_{i}\right)=\left\{ \begin{array}{c} f_{i}^{2}\;i\,f\,y_{i}=2\\ 2\,f_{i}\,\left(1-f_{i}\right)\;i\,f\,y_{i}=1\\ {\left(1-f_{i}\right)}^{2}\;i\,f\,y_{i}=0 \end{array}\right.$$

For a given vector of admixture proportions, the log-likelihood of the observed genotypes *g* for an individual was defined as:

(3) $$L\left(W\right)=\sum_{i=1}^{M}\ln(Pr\left(y_{i}|f_{i}\right)$$

Alternatively, the above likelihood can be written as:

(4) $$L\,\left(W\right)=\sum_{i=1}^{M}\left[y_{i}\,\ln\left(f_{i}\right)+\left(2-y_{i}\right)\,\ln\left(1-f_{i}\right)\right]+C$$

where $C=\sum_{i=1}^{M}l\,n\,\left(\begin{array}{c} 2\\ y_{i} \end{array}\right)$. Given the matrix of allele frequencies *x*_*ij*_ (1≤*i*≤*M*and  1≤*j*≤ K) for *k* populations, our goal was to determine the vector *W* = [*w*_1_, *w*_2_,…,*w*_*K*_]′ of admixture proportions that maximize *L*(*W*) subject to the constraints *w*_*j*_≥0 and $\sum_{j=1}^{K}w_{j}=1$.Optimization of (4), however, is challenged by the constraint on the admixture proportions, that is *w*_*j*_≥0 and $\sum_{j=1}^{K}w_{j}=1$. Alexander et al. (2009) used sequential quadratic programming combined with a quasi-Newton acceleration method to optimize the likelihood function. This method, however, involves the manipulation and inversion of a possibly large matrix, which can be computationally intensive. Following Bansal and Libiger (2015) and He et al. (2018), we utilized the Broyden-Fletcher-Goldfarb-Shanno (BFGS) method to optimize the likelihood function (4). The BFGS algorithm is a popular quasi-Newton method for solving non-linear optimization problems, which utilizes the first derivatives of the likelihood function and approximates the Hessian matrix of the second derivatives (Nocedal and Wright, 2006). The constraint $\sum_{j=1}^{K}w_{j}=1$ is handled by scaling the individual admixture coefficients by their sum, that is, replacing *w*_*j*_ with $\frac{w_{j}}{\sum_{j=1}^{K}w_{j}}$ in the likelihood function.

#### Path Analysis

Intuitively, path analysis can be viewed as an extension of linear regression in the form of standardized multiple regression, yet with a focus on inferring causality (Wright, 1921). By centering *y*_*i*_ and each *x*_*j*_ on zero (i.e., subtracting the expectation of each corresponding variable), and after dividing both sides of equation (1) by the standard deviation of ***y***, the linear regression model can be expressed as:

$$\frac{y_{i}-E\,\left(y_{i}\right)}{\sigma_{y_{i}}}=\sum_{j=1}^{K}\left\{b_{j}\times \frac{x_{j}-E\,\left(x_{j}\right)}{\sigma_{y_{i}}}\right\}+\frac{e_{i}-E\,\left(e_{i}\right)}{\sigma_{y_{i}}}$$

which can be further re-arranged as:

(5) $$\frac{y_{i}-E\,\left(y_{i}\right)}{\sigma_{y_{i}}}=\sum_{j=1}^{K}\left\{b_{j}\times \frac{\sigma_{x_{j}}}{\sigma_{y_{i}}}\times \frac{x_{j}-E\,\left(x_{j}\right)}{\sigma_{x_{j}}}\right\}+\frac{\sigma_{e_{i}}}{\sigma_{y_{i}}}\times \frac{e_{i}-E\,\left(e_{i}\right)}{\sigma_{e_{i}}}$$

Now, Let $y_{i}^{*}=\frac{y_{i}-E\,\left(y\right)}{\sigma_{y_{i}}}$,$x_{j}^{*}=\frac{x_{j}-E\,\left(x_{j}\right)}{\sigma_{x_{j}}}$,$e_{i}^{*}=\frac{e_{i}-E\,\left(e\right)}{\sigma_{e_{i}}}$, $b_{j}^{*}=b_{j}\,\frac{\sigma_{x_{j}}}{\sigma_{y_{i}}}$, and $b_{e}^{*}=1\times \frac{\sigma_{e_{i}}}{\sigma_{y_{i}}}$. Then, the above equation is simplified to be:

(6) $$y_{i}^{*}=\sum_{j=1}^{K}\left\{b_{j}^{*}\times x_{j}^{*}\right\}+b_{e_{i}}^{*}\times e_{i}^{*}$$

Here, $y_{i}^{*}$, $x_{j}^{*}$, and $e_{i}^{*}$ are standardized vectors for genotypes, allele A frequencies, and residuals, respectively, and $b_{j}^{*}=b_{j}\times \left[c\,p\,s\,b\,r\,e\,a\,k\right]\,\frac{\sigma_{x_{j}}}{\sigma_{y_{i}}}$ is a standardized regression coefficient for an exogenous variable, which is also referred to as a path coefficient. That is,

(7) $$p_{y_{i}\,x_{j}}=b_{j}\times \frac{\sigma_{x_{j}}}{\sigma_{y_{i}}}$$

In (6), $b_{e}^{*}$ is the path coefficient pertaining to the residual term, which is also referred to as the coefficient of alienation (Land, 1969). For the estimation of GBC, the presence of this residual term is relevant for two main reasons. Firstly, Mendelian sampling deviates the GBC of individual animals from their expected values. Secondly, the allele frequencies of the ancestral breeds are contemporary, which can be different from those of the base populations when the crossbreeding for creating this composite breed was initiated. Over the years, allele frequencies of the ancestral breeds can change to a varying extent due to selection, migration, and inbreeding. In what follows, we ignore the superscript “^∗^” for the convenience of notation. If we replace the standardized regression coefficients with the path coefficient notation in (6), it gives:

(8) $$y_{i}=\sum_{j=1}^{K}\left\{p_{y_{i}\,x_{j}}\times x_{j}\right\}+p_{y_{i}\,e_{i}}\times e_{i}$$

In the path analysis, a path coefficient measures the fraction of standard deviation of standardized genotypes of a crossbred animal for which each ancestor or ancestral breed is directly responsible, in the sense of the fraction which would be found if the allele frequencies of one ancestral breed varies to the same extent as in the observed data while all other variables (i.e., allele frequencies of the other ancestral breeds) are constant.

The theory of path analysis states that the correlation between y and *x*_*j*_ is the sum of direct path coefficient plus a sum of terms each quantifying a correctional or an indirect path effect:

(9) $$r_{y_{i}\,x_{j}}=p_{y_{i}\,x_{j}}+\sum_{j^{\prime }\neq j}^{K}p_{y_{i}\,x_{j^{\prime }}}\,r_{x_{j}\,x_{j^{\prime }}}$$

Thus, a path coefficient represents the direct effect of an ancestor or ancestral breed to be a cause on the genome of a crossbred animal while the latter is assumed to be an effect, whereas the correlation *r*_*y_i x_j*_ reflects the genomic similarity between them. Then, the determination of an endogenous variable (genotypes of a crossbred animal) on an exogenous variable (allele frequencies of a reference population) is measured by the coefficient of determination. For example, the coefficient of determination of *x*_*j*_ on *y*_*i*_ is given by the sum of the squared direct path coefficient and the terms representing the determination of all possible indirect paths. That is,

(10) $$d_{y_{i}\,x_{j}}=p_{y_{i}\,x_{j}}^{2}+\sum_{j^{\prime }\neq j}^{K}p_{y_{i}\,x_{j}}\,r_{x_{j}\,x_{j^{\prime }}}\,p_{y_{i}\,x_{j^{\prime }}}$$

The above is referred to as the coefficient of combined determination for an exogenous variable (*x*_*j*_), which includes correlational, indirect path effects. When the correlational effects are zero or ignored, the above reduces to the coefficient of direct determination of *x*_*j*_ on *y*_*i*_,

(11) $$d_{y_{i}\,x_{j}}=p_{y_{i}\,x_{j}}^{2}$$

Hence, the coefficient of direct determination of an exogenous variable to the endogenous variable, which is the squared path coefficient, measures the proportion of the variance of the endogenous variable for which an exogenous variable is directly responsible. Then, it can be shown that the total variation of the endogenous variable is entirely determined by a linear combination of the exogenous and the residual variable(s). That is,

(12) $$\sum_{j=1}^{K}\left(p_{y_{i}\,x_{j}}^{2}+\sum_{j^{\prime }\neq j}^{K}p_{y\,x_{j}}\,r_{x_{j}\,x_{j^{\prime }}}\,p_{y_{i}\,x_{j^{\prime }}}\right)+p_{y_{i}\,e_{i}}^{2}=1$$

Thus, in view of genomic determination, GBC can be measured by the relative ratio of the coefficients of either the direct or combined determination. Hereafter, the former is referred to as D-GBC and the latter C-GBC hereafter. That is,

D-GBC$=p_{y_{i}\,x_{j}}^{2}/\sum_{j=1}^{K}p_{y_{i}\,x_{j}}^{2}$ (13)

(13) $$C-GBC=\left(p_{y_{i}x_{j}}^{2}+\sum_{j^{\prime }\neq j}^{K}p_{y_{i}x_{j}}r_{x_{j}x_{j^{\prime }}}p_{y_{i}x_{j^{\prime }}}\right)/\sum_{j=1}^{K}\left(p_{y_{i}x_{j}}^{2}+\sum_{j^{\prime }\neq j}^{K}p_{y_{i}x_{j}}r_{x_{j}x_{j^{\prime }}}p_{y_{i}x_{j^{\prime }}}\right)(14)$$

The sum of GBC for an individual animal is one when using either of the above two formulas. The difference between the above two measures of GBC is that correlational or indirect path effects are included in the estimated C-GBC with (14) but not in the estimated D-GBC with (13). From the viewpoint of genetic determination, the correlational or indirect path effects are attributable to genomic similarities. The proportion of the variance of the endogenous variable that is not accounted for by the set of exogenous variables in the system is then quantified to be:

(14) $$\text{R}=p_{y_{i}\,e_{i}}^{2}=1-\sum_{j=1}^{K}\left(p_{y_{i}\,x_{j}}^{2}+\sum_{j^{\prime }\neq j}^{T}p_{y_{i}\,x_{j}}\,r_{x_{j}\,x_{j^{\prime }}}\,p_{y_{i}\,x_{j^{\prime }}}\right)$$

Note that 1-R can be used as a measure of reliability say for estimated C-GBC. Given two individuals having the same values of C-GBC but different R values, estimated C-GBC is more reliable for the one with a smaller value of 1-R.

In the above, we have discussed the path analysis applied to estimate GBC as a form of standardized linear regression, in which standardized genotypes of each crossbred animal are regressed on standardized allele frequencies of reference breeds. The genotypes are coded as the portion of allele A in the genotypes (i.e., AA = 1, AB = 0.5, and BB = 0), which can also be viewed as the frequency allele A at the individual level. Put in another way, the frequency of allele A at each SNP locus for a given population can be viewed as the average genotype for that population. Another approach is to obtain the path coefficients using the correlations between them, as suggested by the relationships shown in (9). If we extend each equation in (9) for each of the crossbred animals, it gives:

(15) $$\begin{array}{c} p_{y_{i}\,x_{1}}+r_{x_{1}\,x_{2}}\,p_{y_{i}\,x_{2}}+\ldots +r_{x_{1}\,x_{T}}\,p_{y_{i}\,x_{K}}=r_{y_{i}\,x_{1}}\\ \ldots \\ r_{x_{T}\,x_{1}}\,p_{y_{i}\,x_{1}}+r_{x_{T}\,x_{2}}\,p_{y_{i}\,x_{2}}+\ldots +p_{y_{i}\,x_{K}}=r_{y_{i}\,x_{T}} \end{array}$$

where, for example, *r*_*y_i x_1*_ is the correlation between the genotypes of the crossbred animals and the corresponding SNP allele frequencies in the first reference population. In matrix notation, the above becomes:

(16) $$p_{\mathrm{yx}}\,R_{\mathrm{xx}}=r_{\mathrm{yx}}$$

where *p*_yx_ = (*p*_*y*_*i*_*x*_1__… *p*_*yx*_*T*__)′,*r*_yx_ = (*r*_*yx*_1__…*r*_*yx*_*T*__)′, and

(17) $$R_{\mathrm{xx}}=\left[\begin{array}{ccc} \begin{array}{c} 1\\ r_{x_{2}\,x_{1}}\\ \begin{array}{c} \ldots \\ r_{x_{T}\,x_{1}} \end{array} \end{array} & \begin{array}{c} \begin{array}{cc} r_{x_{1}\,x_{2}} & \ldots \end{array}\\ \begin{array}{cc} 1 & \ldots \end{array}\\ \begin{array}{cc} \begin{array}{c} \ldots \\ r_{x_{T}\,x_{2}} \end{array} & \begin{array}{c} \ldots \\ \ldots \end{array} \end{array} \end{array} & \begin{array}{c} r_{x_{1}\,x_{T}}\\ r_{x_{2}\,x_{T}}\\ \begin{array}{c} \ldots \\ 1 \end{array} \end{array} \end{array}\right]$$

Therefore, the vector of path coefficients is obtained as:

(18) $$p_{\mathrm{yx}}=r_{y\,x}\,R_{x\,x}^{-1}$$

Now consider only two exogenous variables,*x*_1_ and *x*_2_. The solutions of the path coefficients are obtained as the following:

(19) $$\begin{array}{c} p_{y_{i}\,x_{1}}=\left(r_{y_{i}\,x_{1}}-r_{x_{1}\,x_{2}}\,r_{y_{i}\,x_{2}}\right)/\left(1-r_{x_{1}\,x_{2}}^{2}\right)\\ p_{y_{i}\,x_{2}}=\left(r_{y_{i}\,x_{2}}-r_{x_{1}\,x_{2}}\,r_{y_{i}\,x_{1}}\right)/\left(1-r_{x_{1}\,x_{2}}^{2}\right) \end{array}\;$$

In the above, *p*_*y_i x_1*_ is also recognized as the semi-partial correlation of *x*_1_ on *y*_*i*_, and *p*_*y_i x_2*_ is the semi-partial correlation of *x*_2_ on *y*_*i*_. Like a partial correlation, a semi-partial correlation compares variations of two variables after certain factors are controlled for. The difference between them is that, with a semi-partial correlation, one holds the third variable (*x*_2_) constant for either *x*_1_ or *y*_*i*_ but not both, whereas with a partial correlation, one holds the third variable constant for both (Baba et al., 2004). In terms of their quantities, the absolute value of a semi-partial correlation, say between *x*_1_ and *y*_*i*_, is always no greater than that of the partial correlation between the two variables. We used Pearson’s correlations of allele A frequencies in the path analysis, though the distributions of allele A frequencies were not exactly normal distributions, but taken to be so approximately. Alternatively, Spearman’s correlations can be used as well, which can better capture monotonic relationships. Nevertheless, relational plots of allele frequencies between breeds showed apparently linear relationships between a composite breed and its ancestral breed, not monotonic relationships. That was another reason for us to use Pearson’s correlations in the present study. As we found later, both types of correlations gave well comparable results.

**FIGURE 1:**
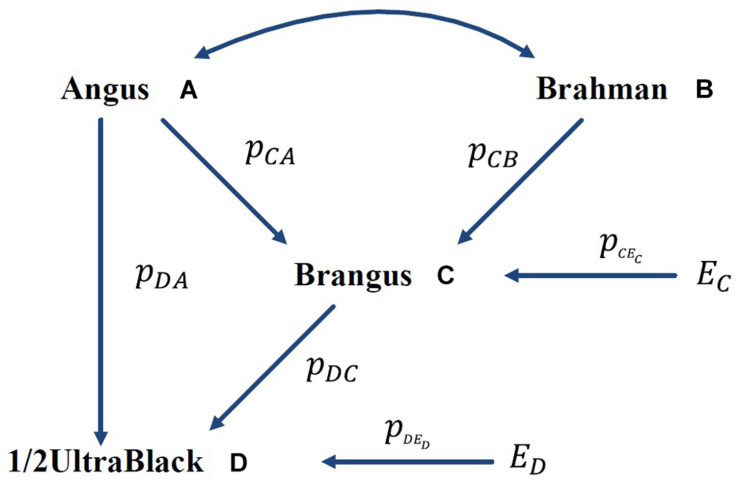
Path diagram of the relationships between Brangus (and 1/2 UltraBlack) and two ancestral breeds, namely Angus and Brahman *p*_*yx*_ = path coefficient from *x* to *y*; *r*_*AB*_ = correlation between Angus (A) and Brahman (B).

A numeric example is shown in Figure 1, where the GBC is computed for an Ultrablack, given the assumed GBC of Brangus. The International Brangus Breeders Association has created an appendix registry designation of Ultrablack (and Ultrared) for animals, which are between 12.5 and 87.5% Brangus and the remainder Angus (or Red Angus) (Waldrip, 2017). For the convenience of discussion, we will use Ultrablack to represent first-generation Ultrablack animals (1/2 Brangus × 1/2 Angus). For the convenience of discussion, we assume that *r*_*AB*_ = 0 (no correlation between Angus and Brahman) and *p*_*CE*_*C*__ = 0 (no residual effect) in this example. Let $p_{C\,A}^{2}=0.625$ and $p_{C\,B}^{2}=0.375$, which is equivalent to a causality interpretation that the Angus origin and Brahman origin accounted for 5/8 (62.5%) and 3/8 (37.5%), respectively, of the genomic variation of Brangus. Then, the GBC of a 1/2 UltraBlack Brangus is computed as follows:

$$d_{D\,A}=p_{D\,A}^{2}+{\left(p_{C\,A}\times p_{D\,C}\right)}^{2}=0.5+{\left(\sqrt{0.625}\times \sqrt{0.5}\right)}^{2}=0.8125$$

$$d_{D\,B}={\left(p_{C\,B}\times p_{D\,C}\right)}^{2}={\left(\sqrt{0.375}\times \sqrt{0.5}\right)}^{2}=0.1875$$

In the above, AD is a direct path from Angus to an Ultrablack, and ACD is an indirect path from Angus to an Ultrablack via Brangus. Similarly, BCD is an indirect path from Brahman to an Ultrablack via Brangus. Note that both indirect paths, ACD and BCD, are two compound paths. The coefficient of a compound path is the product of all component segments. Hence, under the assumption of no correlation between the two ancestral breeds, computed GBC of an Ultrablack agreed with pedigree-expected ratios of genomic composition for an Ultrablack animal, which is 81.25% Angus and 18.75% Brahman. Therefore, assuming no correlation between the two ancestral breeds, the causality interpretation of GBC agreed with the pedigree-expected GBC for an Ultrablack animal.

## Results and Discussion

### Estimated Genomic Breed Composition for Brangus

The Brangus was highly correlated in terms of allelic frequencies with Angus (0.671–0.714) and moderately correlated with Brahman (0.442–0.481) because the Brangus was descended from these two breeds (Table 2). The Brangus animals also had moderate or high correlations with some non-ancestral breeds, such as Simmental (0.585–0.628), Limousine (0.512–0.557), and Shorthorn (0.452–0.520), due to the significant correlations between Angus and these non-ancestral beef breeds. Also based on allelic frequencies, Angus was highly correlated with Gelbvieh (0.765–0.793), Limousine (0.628–0.792), Shorthorn (0.552–0.611), and Simmental (0.776–0.812). The Brahman is a *Bos taurus indicus* cattle breed, and it had low correlations with the seven *Bos taurus Taurus* cattle breeds (0.035–0.239). Thus, a high correlation between the Brangus and a reference breed does not indicate the genomic causality but the genomic similarity between them.

**TABLE 2:** Path analysis using the correlation data for 7,969 Brangus animals with eight reference breeds and three SNP panels (1K, 5K, and 10K).

Statistic	Breed	GGP 30K/GGP 40K			GGP 50K		
		1K	5K	10K	1K	5K	10K
Correlation with Brangus	Angus	0.699	0.671	0.692	0.714	0.689	0.711
	Brahman	0.442	0.451	0.475	0.444	0.456	0.481
	Gelbvieh	0.635	0.606	0.627	0.647	0.622	0.645
	Hereford	0.362	0.316	0.310	0.374	0.325	0.321
	Limousin	0.532	0.512	0.541	0.546	0.527	0.557
	Shorthorn	0.507	0.452	0.478	0.520	0.468	0.495
	Simmental	0.610	0.585	0.611	0.624	0.602	0.628
	Wagyu	0.278	0.311	0.344	0.288	0.318	0.353
Path coefficient	Angus	0.527	0.510	0.539	0.538	0.520	0.552
	Brahman	0.402	0.396	0.404	0.403	0.401	0.407
	Gelbvieh	0.107	0.086	0.071	0.096	0.085	0.073
	Hereford	0.019	0.031	0.008	0.023	0.032	0.009
	Limousin	0.030	0.034	0.030	0.037	0.037	0.031
	Shorthorn	0.087	0.060	0.051	0.091	0.068	0.058
	Simmental	−0.007	0.003	0.007	−0.005	0.006	0.007
	Wagyu	−0.029	0.008	0.003	−0.023	0.007	0.003
D-GBC	Angus	60.2%	60.4%	62.9%	61.4%	60.7%	63.4%
	Brahman	35.2%	36.5%	35.3%	34.4%	36.0%	34.6%
	Gelbvieh	2.48%	1.71%	1.09%	1.94%	1.63%	1.09%
	Hereford	0.07%	0.22%	0.01%	0.11%	0.23%	0.02%
	Limousin	0.20%	0.28%	0.19%	0.29%	0.31%	0.20%
	Shorthorn	1.64%	0.82%	0.56%	1.77%	1.03%	0.71%
	Simmental	0.01%	0.00%	0.01%	0.00%	0.01%	0.01%
	Wagyu	0.18%	0.02%	0.00%	0.12%	0.01%	0.00%
C-GBC	Angus	56.9%	57.1%	56.7%	57.6%	57.2%	57.6%
	Brahman	30.2%	32.2%	30.2%	29.4%	31.5%	29.4%
	Gelbvieh	6.67%	5.13%	6.66%	5.87%	5.11%	5.87%
	Hereford	0.59%	0.92%	0.59%	0.75%	0.95%	0.75%
	Limousin	1.41%	1.60%	1.41%	1.75%	1.75%	1.75%
	Shorthorn	4.34%	2.63%	4.34%	4.63%	3.08%	4.63%
	Simmental	0%	0.16%	0%	0%	0.30%	0%
	Wagyu	0%	0.22%	0%	0%	0.20%	0%

**The Brangus animals were genotyped on either GGP 30K/GGP 40K bovine SNP chip or GGP 50K bovine SNP chip, respectively.**

The path analysis estimated the genomic effects of these reference breeds on the Brangus. With the eight reference breeds, the estimated path coefficients for the two ancestral breeds were the largest among the eight reference breeds, which were 0.510–0.552 for Angus, and 0.396–0.407 for Brahman (Table 2). The D-GBC for the two ancestral breeds was estimated to be 60.2–63.4% (Angus) and 34.4–36.5% (Brahman), and the C-GBC for the two ancestral breeds, which included both direct and indirect path effects, was estimated to be 57.1–58.6% (Angus) and 29.6–32.2% (Brahman) (Table 2). It is noted that, with the eight reference breeds, the estimated D-GBC and C-GBC for the two ancestral breeds were both considerably below the pedigree-expected ratios of 62.5% for Angus origin and 37.5% for Brahman origin, regardless of the genotyping platforms and SNP panels used. Hence, by including non-ancestral reference breeds, it introduced noise (i.e., small estimated GBC for non-ancestral breeds) in the estimation of GBC for the Brangus, which in turn offset to a varying extent the estimated GBC for the ancestral breeds. The estimated D-GBC for non-ancestral breeds was mostly less than 1%, but the estimated C-GBC for non-ancestral was large, which for example, was 5.11–6.72% for Gelbvieh and 2.63–4.67% for Shorthorn, and 1.41–1.77% for Limousine (Table 2). Estimated D-GBC and C-GBC by the path analysis using the genotype data showed similar patterns (Supplementary Table 1). Therefore, when the eight reference cattle breeds were used, the small amounts of estimated GBC for non-ancestral breeds offset the estimated GBC for the ancestral breeds, thus leading to underestimated GBC for the ancestral breeds, regardless of the models used.

The bias in the estimated GBC can be minimized by excluding non-ancestral breeds from the reference breed panel based on *a priori* information. Because Brangus cattle are descended from Angus and Brahman, we estimated D-GBC and C-GBC by including only the two ancestral breeds as the reference breeds. Then, with these two reference breeds only, the estimated D-GBC for the Brangus using the correlation data was 71.2–72.0% Angus and 28.0–28.8% Brahman, and the estimated C-GBC for the Brangus was 70.2–71.2% Angus and 28.7–29.8% Brahman (Table 3). Pearson’s correlations were used by path analysis throughout the present study, though the allele frequencies of the SNPs were not exactly normal distributions. Switching to using Spearman’s correlations, for example, led to slightly different results, but they were well comparable to the results obtained based on Pearson’s correlations. For example, based on the Spearman’s correlations of allele A frequencies, the estimated D-GBC was 69.4–74.1% of Angus and 25.9–30.6% of Brahman. These values are within a comparable range of those obtained based on Pearson’s correlations of allele A frequencies. The estimated D-GBC from the path analysis using the genotype data was 69.5–71.8% Angus and 28.2–30.5% Brahman, respectively, and the estimated C-GBC were 68.2–70.9% Angus and 29.1–31.8% Brahman (Table 4). With the genotype data, the admixture model suggested that the Brangus were on average 68.8–70.3% Angus and 29.7–31.2% Brahman, whereas the linear regression indicated that Brangus was 68.6–70.4% Angus and 29.6–31.4% Brahman (Table 4). The estimated D-GBC and the estimated C-GBC for the Brangus in the path analysis agreed approximately with each other when the correlation in allelic frequencies between the two ancestral breeds was low (0.051–0.090). In other words, the correlational indirect path effects between the ancestral breeds are trivial, and thus the estimated D-GBC agreed well with the estimated C-GBC. The estimated D-GBC and C-GBC from the path analysis also corresponded well to the estimated GBC from the admixture model and linear regression in this Brangus population (Table 4). It also came to our attention that the estimated GBC did not show significant differences between different genotyping platforms and between three SNP panels used (Tables 3, 4).

**TABLE 3:** Path analysis using the correlation data for 7,969 Brangus animals with two ancestral breeds (Angus and Brahman) as the reference and three SNP panels (1K, 5K, and 10K).

Statistics		GGP30K/GGP 40K			GGP 50K		
		1K	5K	10K	1K	5K	10K
Correlation	Brangus vs. Angus	0.699	0.671	0.692	0.714	0.689	0.711
	Brangus vs. Brahman	0.442	0.451	0.475	0.444	0.456	0.481
Path coefficient	Brangus < -Angus	0.668	0.645	0.654	0.678	0.663	0.673
	Brangus < -Brahman	0.418	0.410	0.416	0.424	0.415	0.420
D-GBC	Brangus < -Angus	71.9%	71.2%	71.2%	71.9%	71.8%	72.0%
	Brangus < -Brahman	28.1%	28.8%	28.8%	28.1%	28.2%	28.0%
C-GBC	Brangus < -Angus	71.2%	70.6%	70.2%	71.2%	71.1%	70.9%
	Brangus < -Brahman	28.7%	29.3%	29.8%	28.7%	28.9%	29.1%

**The Brangus animals were genotyped on either GGP 30K/GGP 40K bovine SNP chip or GGP 50K bovine SNP chip, respectively. The correlation of allele A frequencies between Angus (A) and Brahman (B) computed for the three SNP panels was *r*_*AB*_ = 0.051 (1K), 0.062 (5K) and 0.090 (10K).**

**TABLE 4:** Comparison of estimated GBC for 7,969 Brangus with genotype data, obtained by the admixture model, linear regression, and path analysis techniques, respectively, using only Angus and Brahman in the reference breed set.

Model	Panel	GGP 30K/GGP 40K				GGP 50K			
		Angus		Brahman		Angus		Brahman	
		Mean	*SD*	Mean	*SD*	Mean	*SD*	Mean	*SD*
Admixutre	1K	69.9%	7.3%	30.1%	7.3%	70.3%	7.1%	29.7%	7.1%
	5K	69.8%	6.8%	30.2%	6.8%	70.1%	6.8%	29.9%	6.8%
	10K	68.8%	7.1%	31.2%	7.1%	69.1%	7.0%	30.9%	7.0%
Linear regression	1K	70.0%	7.6%	30.0%	7.6%	70.4%	7.6%	29.6%	7.6%
	5K	69.5%	7.4%	30.5%	7.4%	69.8%	7.5%	30.2%	7.5%
	10K	68.6%	7.5%	31.4%	7.5%	69.0%	7.6%	31.0%	7.6%
Path analysis (D-GBC)	1K	71.8%	11.9%	28.2%	11.9%	71.5%	12.3%	28.5%	12.3%
	5K	69.6%	11.8%	30.4%	11.8%	70.2%	12.4%	29.8%	12.4%
	10K	69.5%	11.7%	30.5%	11.7%	70.2%	12.3%	29.8%	12.3%
Path analysis (C-GBC)	1K	70.9%	11.7%	29.1%	11.7%	70.6%	12.1%	29.4%	12.1%
	5K	68.7%	11.5%	31.3%	11.5%	69.3%	12.0%	30.7%	12.0%
	10K	68.2%	11.3%	31.8%	11.3%	68.8%	11.8%	31.2%	11.8%

**FIGURE 2:**
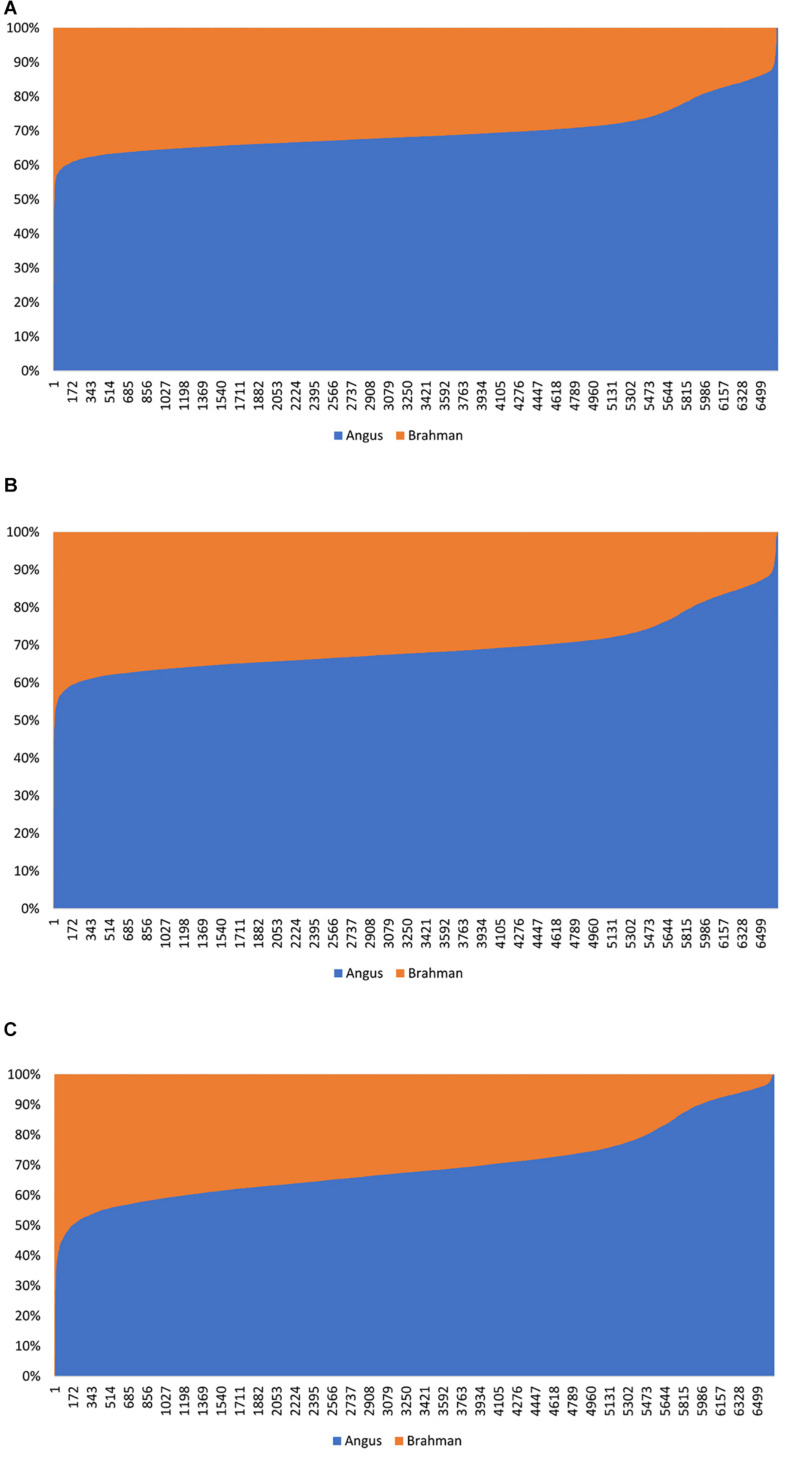
Distribution of estimated genomic breed composition for 7,969 Brangus animals in ascending order of their Angus composition, obtained using three statistical methods: **(A)** admixture model, **(B)** linear regression, and **(C)** path analysis (D-GBC).

The estimated Angus compositions for these Brangus animals by the three methods were all considerably higher than the pedigree-expected Angus ratio (5/8 = 0.625) in Brangus. In the path analysis using the correlation data, for example, the average of estimate Angus GBC was 71.67% across the three genotyping platforms and the three SNP panels. There are possibly two reasons for the elevated Angus composition in these Brangus animals. Firstly, Brangus animals have been selected toward Angus type phenotypes for years, which in turn could have left up the Angus genomic composition in Brangus. Secondly, these Brangus animals included some UB individuals. The estimated GBC for these 7,696 Brangus animals was plotted in ascending order of their Angus composition (Figure 2). The mixture of the UB animals was identified by the sharp increase of Angus GBC on the right-hand side of the plot, which roughly accounted for up to one-fourth of the Brangus animals. By roughly taking the portion of 1/2 UB animals to be 25%, which have an average of 81.25% Angus composition, we estimated that the actual Angus composition of the Brangus (non-UB) animals could be (71.67%−81.25%*0.25)/0.75 = 68.5%.

### Estimated Genomic Breed Composition for Beefmaster

The Beefmaster was highly correlated with the three ancestral breeds: Brahman (0.544–0.570), Hereford (0.504–0.549), and Shorthorn (0.443–0.558) (Table 5). There were also moderate to high correlations (0.396–0.551) between the Beefmaster and some non-ancestral beef breeds (e.g., Gelbvieh, Limousin, and Simmental) (Table 5), which arose from the genomic similarities between the ancestral breeds and the non-ancestral breeds. A moderate to a high correlation in allelic frequencies between the Beefmaster and a reference breed was no indication of the genomic causality, but the genomic similarity between them. Of the three ancestral breeds, the correlation was low between Brahman and Hereford (0.035–0.059) and between Brahman and Shorthorn (0.052–0.10), but it was moderate to high between Hereford and Shorthorn (0.381–0.428).

**TABLE 5:** Path analysis using the correlation data for 7,605 Beefmaster animals with eight reference breeds and three SNP panels (1K, 5K, and 10K).

Statistic	Breed	GGP 30K/GGP 40K			GGP 50K		
		1K	5K	10K	1K	5K	10K
Correlation with Beefmaster	Angus	0.384	0.339	0.385	0.436	0.381	0.434
	Brahman	0.552	0.549	0.556	0.544	0.561	0.570
	Gelbvieh	0.477	0.450	0.477	0.551	0.486	0.521
	Hereford	0.511	0.504	0.504	0.549	0.548	0.543
	Limousin	0.441	0.396	0.437	0.528	0.432	0.479
	Shorthorn	0.485	0.443	0.483	0.558	0.477	0.520
	Simmental	0.454	0.415	0.452	0.526	0.455	0.496
	Wagyu	0.367	0.361	0.376	0.435	0.356	0.377
Path coefficient	Angus	−0.008	−0.030	−0.005	0.011	−0.007	0.023
	Brahman	0.498	0.495	0.501	0.522	0.509	0.513
	Gelbvieh	0.040	0.066	0.042	0.047	0.059	0.047
	Hereford	0.347	0.345	0.342	0.380	0.379	0.363
	Limousin	0.015	0.018	0.016	0.041	0.025	0.026
	Shorthorn	0.230	0.216	0.227	0.245	0.228	0.235
	Simmental	0.028	0.024	0.027	0.020	0.033	0.029
	Wagyu	0.041	0.058	0.042	0.023	0.027	0.011
D-GBC	Angus	0.02%	0.22%	0.01%	0.03%	0.01%	0.12%
	Brahman	58.3%	58.3%	59.2%	56.5%	56.2%	57.9%
	Gelbvieh	0.37%	1.02%	0.42%	0.47%	0.76%	0.49%
	Hereford	28.3%	28.3%	27.5%	30.0%	31.3%	29.0%
	Limousin	0.05%	0.07%	0.06%	0.34%	0.13%	0.15%
	Shorthorn	12.4%	11.1%	12.2%	12.4%	11.3%	12.1%
	Simmental	0.19%	0.13%	0.17%	0.08%	0.23%	0.19%
	Wagyu	0.39%	0.80%	0.41%	0.11%	0.16%	0.03%
C-GBC	Angus	0%	0%	0%	0%	0%	0%
	Brahman	44.9%	51.3%	44.9%	42.6%	48.8%	42.3%
	Gelbvieh	3.54%	3.00%	3.54%	4.82%	27.4%	4.82%
	Hereford	33.6%	28.5%	33.6%	35.9%	31.2%	35.9%
	Limousin	0%	0.63%	0%	0%	0.95%	0%
	Shorthorn	15.5%	13.5%	15.5%	15.9%	14.1%	15.6%
	Simmental	0%	0.89%	0%	0%	1.34%	0%
	Wagyu	2.41%	2.15%	2.41%	1.11%	0.86%	1.11%

**The Beefmaster animals were genotyped on either GGP 30K/GGP 40K bovine SNP chip or GGP 50K bovine SNP chip, respectively.**

**FIGURE 3:**
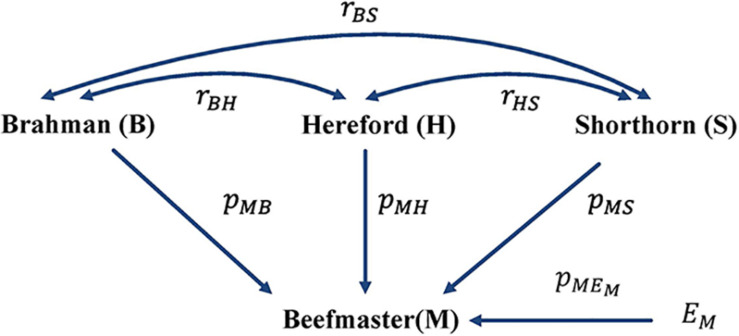
Path diagram of the relationships between Beefmaster and three ancestral breeds, namely Brahman, Hereford, and Shorthorn. *p*_*yx*_ = path coefficient from *x* to *y*; *r*_*xy*_ = correlation between *x* and *y*.

With the correlation data and the eight reference breeds, the path analysis gave the largest estimates of direct path coefficients to the three ancestral breeds, which were 0.495–0.522 (Brahman), 0.342–0.380 (Hereford), and 0.216–0.245 (Shorthorn). Accordingly, the estimated D-GBC for the Beefmaster was 56.2–59.2% (Brahman), 27.5–31.3% (Hereford), and 11.1–12.4% (Shorthorn), whereas the estimated C-GBC for the Beefmaster was 42.6–51.3% (Brahman), 28.5–35.9% (Hereford), and 13.5–15.6% (Shorthorn). Like in the case of Brangus, with the eight reference breeds, estimated GBCs for the ancestral breeds were offset by the small GBC components for non-ancestral breeds (Table 5). The estimated GBC of non-ancestral breeds in the Beefmaster were mostly less than 1% for D-GBC and all below 5% for C-GBC. Estimated D-GBC varied only between different data types, and genotyping platforms, and between the three SNP panels used. The same was true with estimated C-GBC (Table 5). These conclusions coincided with what we had with the Brangus. When limited to three ancestral breeds (Brahman, Hereford, Shorthorn) as the reference, the estimated D-GBC agreed roughly with the estimated C-GBC for the Beefmaster. The estimated D-GBC was 51.3–55.6% (Brahman), 28.6–33.0% (Hereford), and 14.5–17.2% (Shorthorn), whereas the estimated C-GBC was 47.6–51.3% (Brahman), 29.8–34.1% (Hereford), and 17.3–20.6% (Shorthorn) (Table 6). The differences between the estimated D-GBC and the estimated C-GBC for Beefmaster were relatively larger than those observed for Brangus. Similar discrepancies were observed in the results obtained from the path analysis with the genotype data (Table 7). In Beefmaster, the discrepancies between the estimated D-GBC and the estimated C-GBC in the path analysis arose from some significant correlations in allelic frequencies between the ancestral breeds (e.g., between Hereford and Shorthorn). In general, the estimated C-GBC included correlational indirect path effects, but the estimated D-GBC included direct path effects only. The impact of correlations in allelic frequencies between the ancestral breeds on the estimated C-GBC is explained analytically as follows. In Figure 3 is the path diagram for the relationships between the Beefmaster and the three ancestral breeds. Let $p_{M\,B}^{2}=2\,p^{2}$,$p_{M\,H}^{2}=p^{2}$, and $p_{M\,S}^{2}=p^{2}$. Proportionally, the relative direct genomic determination of the three ancestral breeds on the Beefmaster is 50% Brahman, 25% Hereford, and 25% Shorthorn. Thus, when assuming zero correlations between the ancestral breeds and no residual effects, the estimated C-GBC is the same as the estimated D-GBC: 50% Brahman, 25% Hereford, and 25% Shorthorn. However, with non-zero correlations between the ancestral breeds, estimated C-GBC can deviate substantially from estimated D-GBC. For example, let *r*_*BH*_ = 0.10,*r*_*BS*_ = 0.05, and *r*_*HS*_ = 0.40. The estimated C-GBC for each ancestral breed is computed to be a relative ratio of combined determination coefficients for each ancestral breed:

$$C-G\,B\,C_{M\,B}=$$

$$\frac{2\,p^{2}+r_{B\,H}\times \sqrt{2\,p^{2}}\times \sqrt{p^{2}}+r_{B\,S}\times \sqrt{2\,p^{2}}\times \sqrt{p^{2}}}{2\,p^{2}+p^{2}+p^{2}+2\,r_{B\,H}\times \sqrt{2\,p^{2}}\times \sqrt{p^{2}}+2\,r_{H\,S}\times \sqrt{p^{2}}\times \sqrt{p^{2}}+2\,r_{B\,S}\times \sqrt{2\,p^{2}}\times \sqrt{p^{2}}}$$

$$=\frac{2\,p^{2}+0.10\times \sqrt{2}\times p^{2}+0.05\times \sqrt{2}\times p^{2}}{2\,p^{2}+p^{2}+p^{2}+2\times 0.10\times \sqrt{2}\times p^{2}+2\times 0.4\times p^{2}+2\times 0.05\times \sqrt{2}\times p^{2}}$$

$$=\frac{2.212\,p^{2}}{5.224\,p^{2}}=0.423$$

$$C-G\,B\,C_{M\,H}=\frac{p^{2}+r_{B\,H}\times \sqrt{2\,p^{2}}\times \sqrt{p^{2}}+r_{H\,S}\times \sqrt{p^{2}}\times \sqrt{p^{2}}}{2\,p^{2}+p^{2}+p^{2}+2\,r_{B\,H}\times \sqrt{2\,p^{2}}\times \sqrt{p^{2}}+2\,r_{H\,S}\times \sqrt{p^{2}}\times \sqrt{p^{2}}+2\,r_{B\,S}\times \sqrt{2\,p^{2}}\times \sqrt{p^{2}}}$$

$$=\frac{p^{2}+0.10\times \sqrt{2}\times p^{2}+0.4\times p^{2}}{2\,p^{2}+p^{2}+p^{2}+2\times 0.10\times \sqrt{2}\times p^{2}+2\times 0.4\times p^{2}+2\times 0.05\times \sqrt{2}\times p^{2}}$$

$$=\frac{1.541\,p^{2}}{5.224\,p^{2}}=0.295$$

$$C-G\,B\,C_{M\,S}=\frac{p^{2}+r_{B\,S}\times \sqrt{2\,p^{2}}\times \sqrt{p^{2}}+r_{H\,S}\times \sqrt{p^{2}}\times \sqrt{p^{2}}}{2\,p^{2}+p^{2}+p^{2}+2\,r_{B\,H}\times \sqrt{2\,p^{2}}\times \sqrt{p^{2}}+2\,r_{H\,S}\times \sqrt{p^{2}}\times \sqrt{p^{2}}+2\,r_{B\,S}\times \sqrt{2\,p^{2}}\times \sqrt{p^{2}}}$$

$$=\frac{p^{2}+0.05\times \sqrt{2}\times p^{2}+0.4\times p^{2}}{2\,p^{2}+p^{2}+p^{2}+2\times 0.10\times \sqrt{2}\times p^{2}+2\times 0.4\times p^{2}+2\times 0.05\times \sqrt{2}\times p^{2}}$$

$$=\frac{1.571\,p^{2}}{5.224\,p^{2}}=0.282$$

**TABLE 6:** Path analysis using the correlation data for 7,605 Beefmaster animals with three ancestral breeds as the reference and three SNP panels.

Statistics		GGP30K/GGP 40K			GGP 50K		
		1K	5K	10K	1K	5K	10K
Correlation with Beefmaster	Brahman	0.552	0.549	0.556	0.544	0.561	0.570
	Hereford	0.511	0.504	0.504	0.549	0.548	0.543
	Shorthorn	0.485	0.443	0.483	0.558	0.477	0.520
Path coefficient	Brahman	0.513	0.514	0.517	0.536	0.522	0.526
	Hereford	0.375	0.381	0.371	0.420	0.417	0.398
	Shorthorn	0.275	0.263	0.276	0.310	0.282	0.298
D-GBC	Brahman	54.9%	55.2%	55.6%	51.3%	51.9%	52.8%
	Hereford	29.3%	30.3%	28.6%	31.5%	33.0%	30.2%
	Shorthorn	15.7%	14.5%	15.8%	17.2%	15.2%	16.9%
C-GBC	Brahman	50.1%	51.3%	51.0%	46.0%	47.6%	48.0%
	Hereford	31.1%	31.5%	29.8%	33.4%	34.1%	31.4%
	Shorthorn	18.8%	17.3%	19.2%	20.6%	18.3%	20.6%

**The Beefmaster animals were genotyped on either GGP 30K/GGP 40K bovine SNP chip or GGP 50K bovine SNP chip, respectively. Correlations of allele frequencies were 0.059 (1K), 0.045 (5K) and 0.035 (10K) between Brahman and Hereford, 0.052 (1K), 0.069 (5K) and 0.100 (10K) between Brahman and Shorthorn, and 0.460 (1K), 0.381 (5K) and 0.428 (10K) between Hereford and Shorthorn.**

**TABLE 7:** Comparison of estimated GBC for 7,605 Beefmaster animals with genotype data, obtained by the admixture model, linear regression, and path analysis techniques, respectively.

Model	Panel	GGP 30K/GGP 40K						GGP 50K					
		Brahman		Hereford		Shorthorn		Brahman		Hereford		Shorthorn	
		Mean	*SD*	Mean	*SD*	Mean	*SD*	Mean	*SD*	Mean	*SD*	Mean	*SD*
Admixutre	1K	35.9%	4.2%	37.3%	5.6%	26.8%	5.8%	34.2%	4.7%	38.0%	6.0%	27.8%	6.8%
	5K	35.4%	3.3%	36.0%	3.3%	28.5%	3.7%	34.1%	3.9%	37.0%	3.4%	29.0%	4.9%
	10K	36.3%	3.3%	34.8%	3.2%	28.9%	3.7%	35.2%	4.0%	35.3%	3.2%	29.5%	4.8%
Linear regression	1K	36.4%	4.7%	38.0%	6.1%	25.6%	6.1%	34.7%	5.4%	38.8%	6.6%	26.5%	7.7%
	5K	36.8%	3.7%	36.2%	3.9%	27.0%	4.1%	35.2%	4.4%	37.4%	3.8%	27.4%	5.5%
	10K	37.4%	3.7%	35.0%	3.6%	27.6%	4.1%	36.1%	4.5%	35.6%	3.6%	28.3%	5.5%
Path analysis (D-GBC)	1K	50.7%	9.8%	34.9%	10.4%	14.4%	7.2%	47.0%	11.1%	36.9%	11.1%	16.1%	10.9%
	5K	54.7%	7.8%	30.3%	6.8%	15.0%	5.8%	51.1%	9.3%	32.8%	6.7%	16.0%	8.6%
	10K	54.9%	7.7%	28.7%	6.2%	16.3%	6.2%	52.2%	9.5%	30.0%	6.2%	17.7%	9.1%
Path analysis (C-GBC)	1K	43.2%	9.0%	37.0%	8.5%	19.8%	6.8%	39.9%	9.9%	38.7%	9.0%	21.3%	9.5%
	5K	47.5%	7.3%	32.4%	5.8%	20.0%	5.2%	44.3%	8.4%	34.6%	5.6%	21.0%	7.4%
	10K	46.9%	7.1%	30.8%	5.3%	22.2%	5.4%	44.5%	8.4%	32.0%	5.1%	23.5%	7.5%

Therefore, with non-zero correlations between the three ancestral breeds as the reference, in particular when one or more of the correlations are large, estimated C-GBC would deviate considerably from the estimated D-GBC. Generally speaking, the larger the correlation between the ancestral breeds, the larger the deviation that it will generate.

**FIGURE 4:**
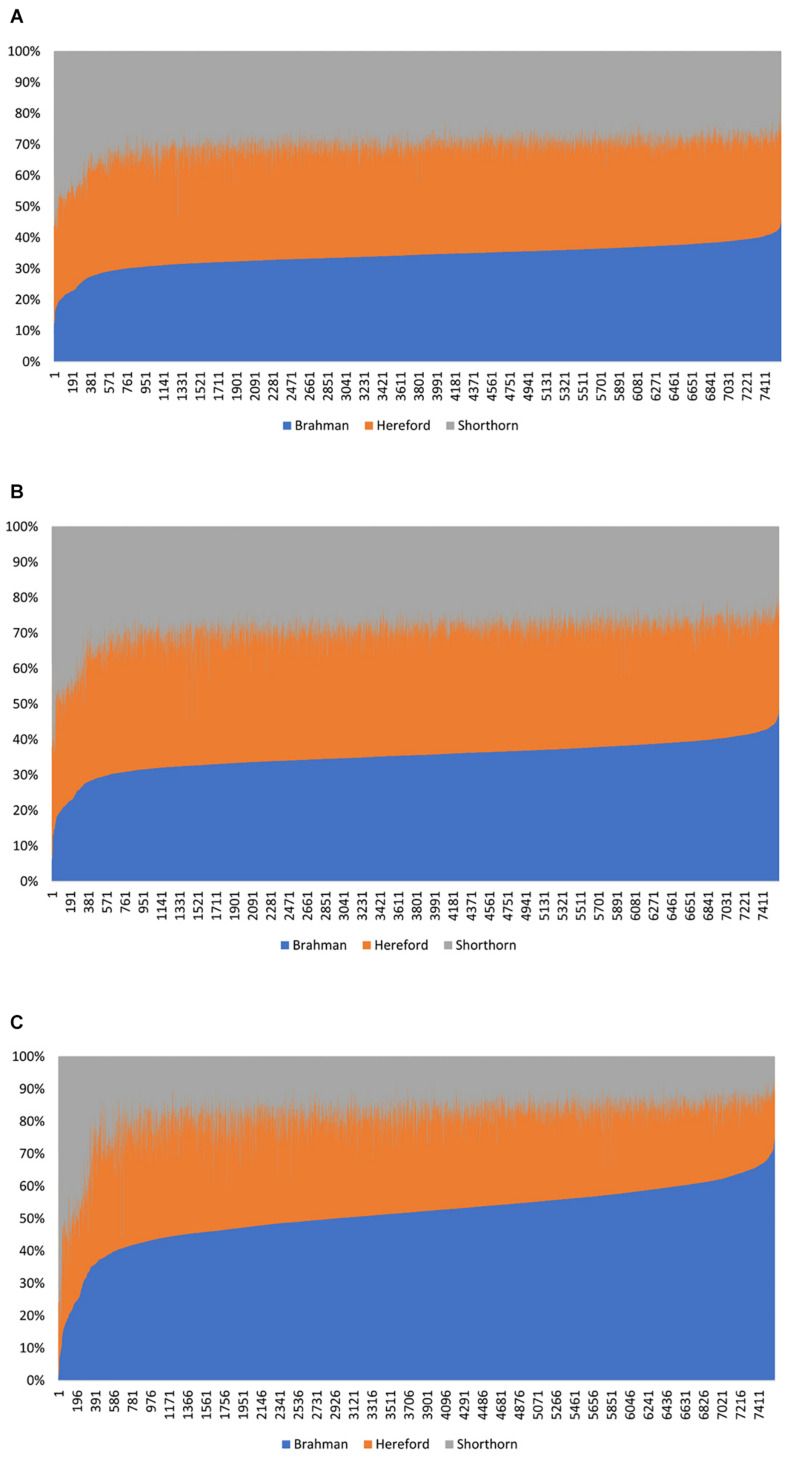
Distribution of the estimated genomic breed composition for 7,605 Beefmaster animals in ascending order of their Brahman composition, obtained using three statistical methods: **(A)** admixture model, **(B)** linear regression, and **(C)** path analysis (D-GBC).

In Beefmaster, the estimated GBCs from the admixture model and the linear regression approach seemed to deviate substantially from pedigree-expected values (i.e., 50% Brahman, 25% Hereford, and 25% Shorthorn). They did not correspond to those obtained from the path analysis neither. The estimated GBC of the Beefmaster obtained from the admixture model was 34.1–36.3% (Brahman), 34.8–38.0% (Hereford), and 26.8–29.5% (Shorthorn). The estimated GBC of the Beefmaster obtained from the linear regression was 34.7–37.4% Brahman, 35.0–38.8% Hereford, and 25.6–28.3% Shorthorn. Relatively speaking, the estimated GBC from the admixture model and the linear regression were closer to the estimated C-GBC than the estimated D-GBC, possibly because they all included correlational indirect path effects except the estimated D-GBC. The distributions of the estimated GBC for 7.605 Beefmaster animals in ascending order of the Brahman composition obtained using the three statistical models are shown in Figure 4.

In the linear regression approach, high correlations between exogenous variables translate into strong multicollinearity, which imposes some challenges to the identification of the likelihood in the admixture model. The problem of model identification may not necessarily affect the prediction accuracy, but individual parameters can be unidentified and cannot be estimated uniquely or reliably. Similarly, high correlations between exogenous variables can bring challenges for the admixture model to precisely assess the weights for the underlying admixture components, which in the admixture model were the allele frequencies of ancestral breeds as random variables. Arguably, the linear regression approach and the admixture model are not appropriate for estimating GBC when the ancestral breeds are highly correlated. Instead, estimated D-GBC from the path analysis are robust to deviations due to correlational path effects.

## Conclusion

We proposed a causality interpretation of genomic breed composition implemented by the path analysis for composite animals in the present study. Two measures of GBC using path analysis were proposed in the present study. Of them, D-GBC considered only direct path effects of each reference breed, whereas C-GBC also included indirect path effects due to the correlation between reference breeds. In Brangus, because the two ancestors breeds are remotely related, or they have a close to zero correlation, the estimated D-GBC agreed with the estimated C-GBC in the path analysis, and they both agreed well with the estimated GBC by the admixture model and linear regression. However, when the ancestors are highly correlated, which was the case with Beefmaster, the estimated D-GBC showed relatively larger differences from the estimated C-GBC in the path analysis because the latter included correlational effects due to genomic similarity between ancestors. Relatively speaking, the estimated GBC from the admixture model and linear regression were closer to the estimated C-GBC by path analysis than the estimated D-GBC. A possible reason is that the estimated GBC from the admixture model and linear regression (and C-GBC by path analysis) included correlational effects. Thus, path analysis provides an alternative interpretation and an estiamation method of GBC, which arguably has advantages when reference (ancestral) breeds are highly genetically correlated. Finally, estimated GBC varied only slightly between different genotyping platforms (30K/40K vs. 50K) and between the three SNP panel sizes (1K, 5K, and 10K) when subsets consisted of uniformly distributed SNPs.

## Data Availability Statement

All data were taken from the databases of the Neogen Global Laboratories. Example data and programs are available at: https://drive.google.com/drive/folders/1Vs3H-oKc5A9xmX1MU2juuI8gC925wLny?usp=sharing. Further inquiries can be directed to the corresponding author.

## Author Contributions

X-LW, RT, GR, and SB conceived and designed this study, with discussions participated by RF, JG, JP, and TS. X-LW, ZL, JH, and YW carried out the analyses. X-LW drafted this manuscript. All authors have reviewed and approved the final manuscript.

## Conflict of Interest

X-LW, RF, JG, RT, JP, TS, and SB were employees of the Neogen GeneSeek, an agricultural genomics service company. The remaining authors declare that the research was conducted in the absence of any commercial or financial relationships that could be construed as a potential conflict of interest.

## Supplementary Material

Supplementary Figure 1 Map view of three SNP panels used in the present study: **(A)** 10K SNP panel, **(B)** 5K SNP panel, and **(C)** 1K SNP panel.

Supplementary Figure 2 Hierarchical clustering of eight reference breeds used in the present study as the reference.

Supplementary Figure 3 Histograms of SNP allele A frequencies in eight reference breeds and two composite breeds.

Supplementary Table 1 Path analysis for 7,969 Brangus animals using the genotype data, and evaluated by three SNP panels (1K, 5K, and 10K). The putative ancestral reference breeds consisted of eight reference beef breeds. The Brangus animals were genotyped on either GGP 30K/GGP 40K bovine SNP chip or GGP 50K bovine SNP chip, respectively.
