# Supplementary material for: A Causality Perspective of Genomic Breed Composition for Composite Animals
Source: Front Genet. 2020 Oct 30;11:546052. doi: 10.3389/fgene.2020.546052 (PMC7662449; doi:10.3389/fgene.2020.546052)
Supplement: Supplementary Figure 1 — Map view of three SNP panels used in the present study: (A) 10K SNP panel, (B) 5K SNP panel, and (C) 1K SNP panel. [file Data_Sheet_1.PDF]

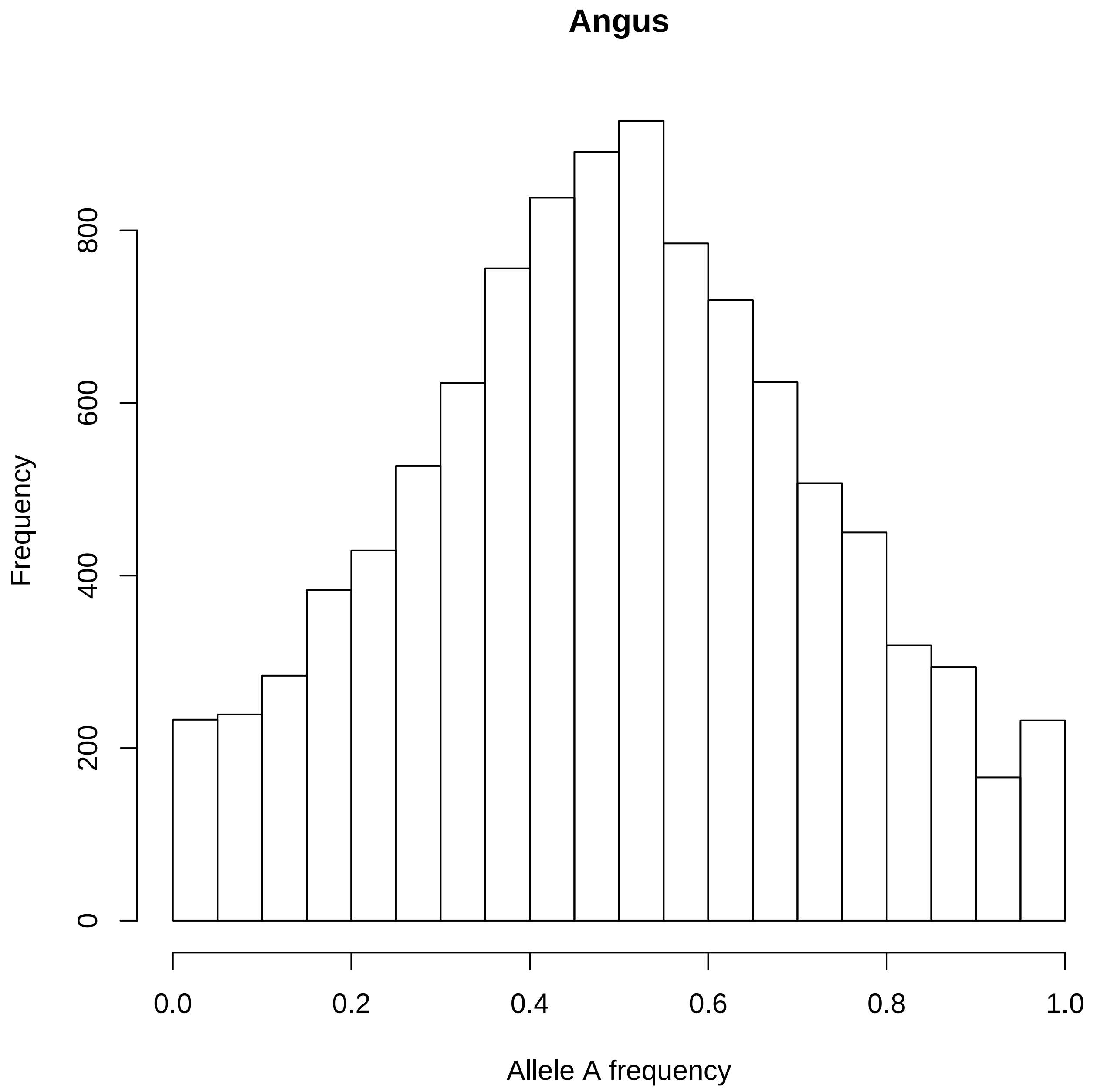
Angus
Frequency
0
200
400
600
800
0.0
0.2
0.4
0.6
0.8
1.0
Allele A frequency

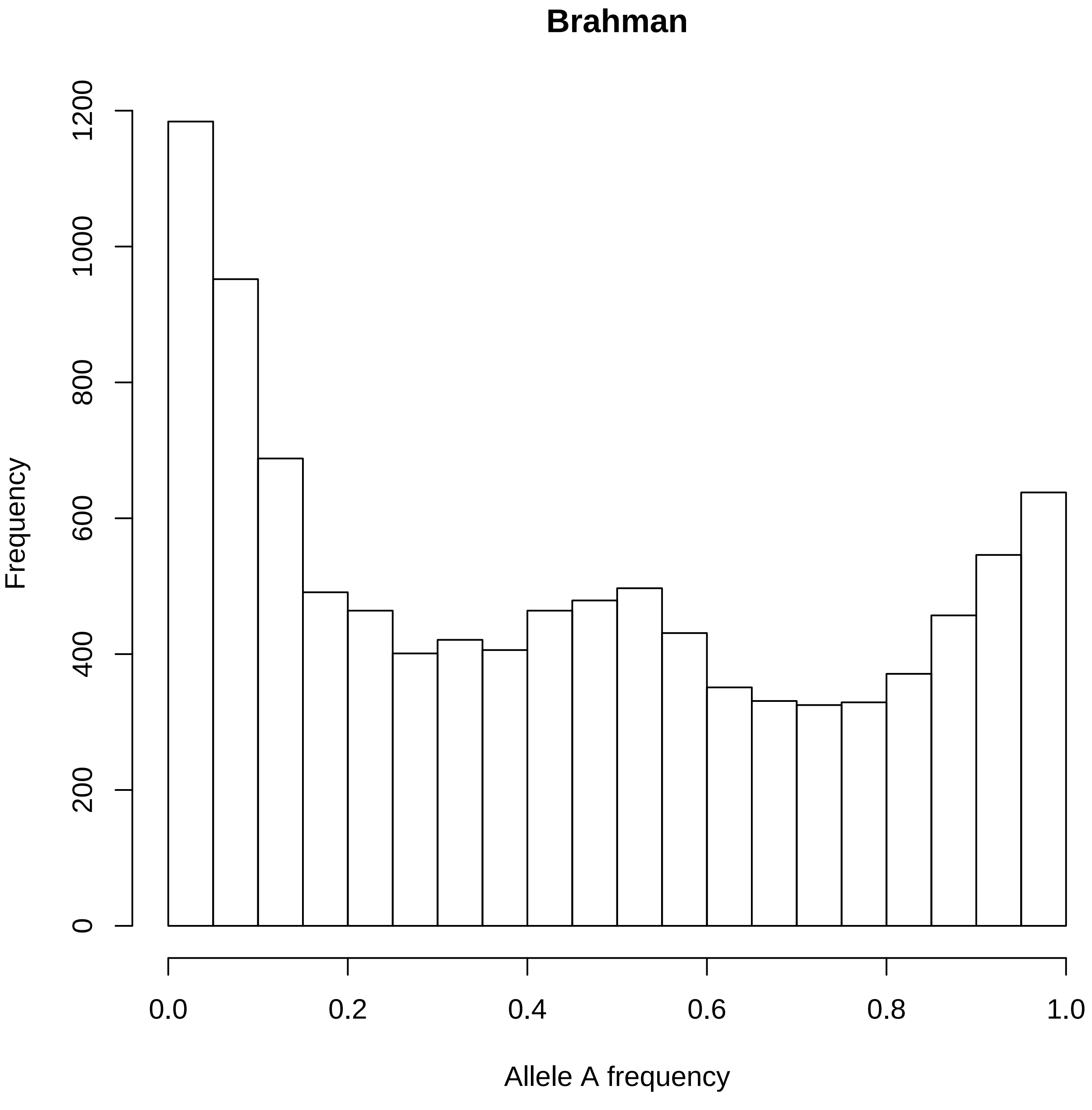
Brahman
Frequency
0
200
400
600
800
1000
1200
0.0
0.2
0.4
0.6
0.8
1.0
Allele A frequency

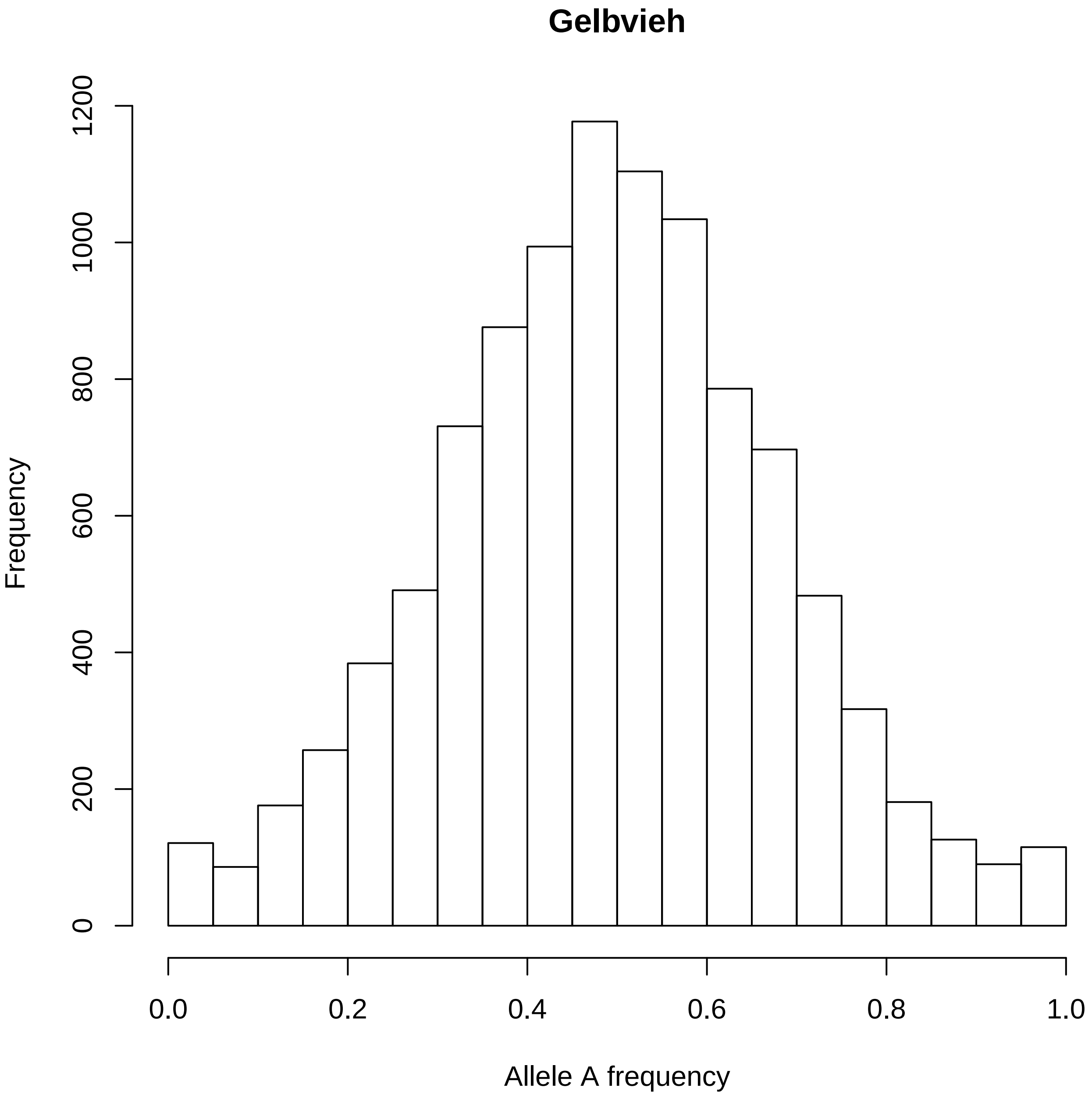
Gelbvieh
Frequency
0
200
400
600
800
1000
1200
0.0
0.2
0.4
0.6
0.8
1.0
Allele A frequency

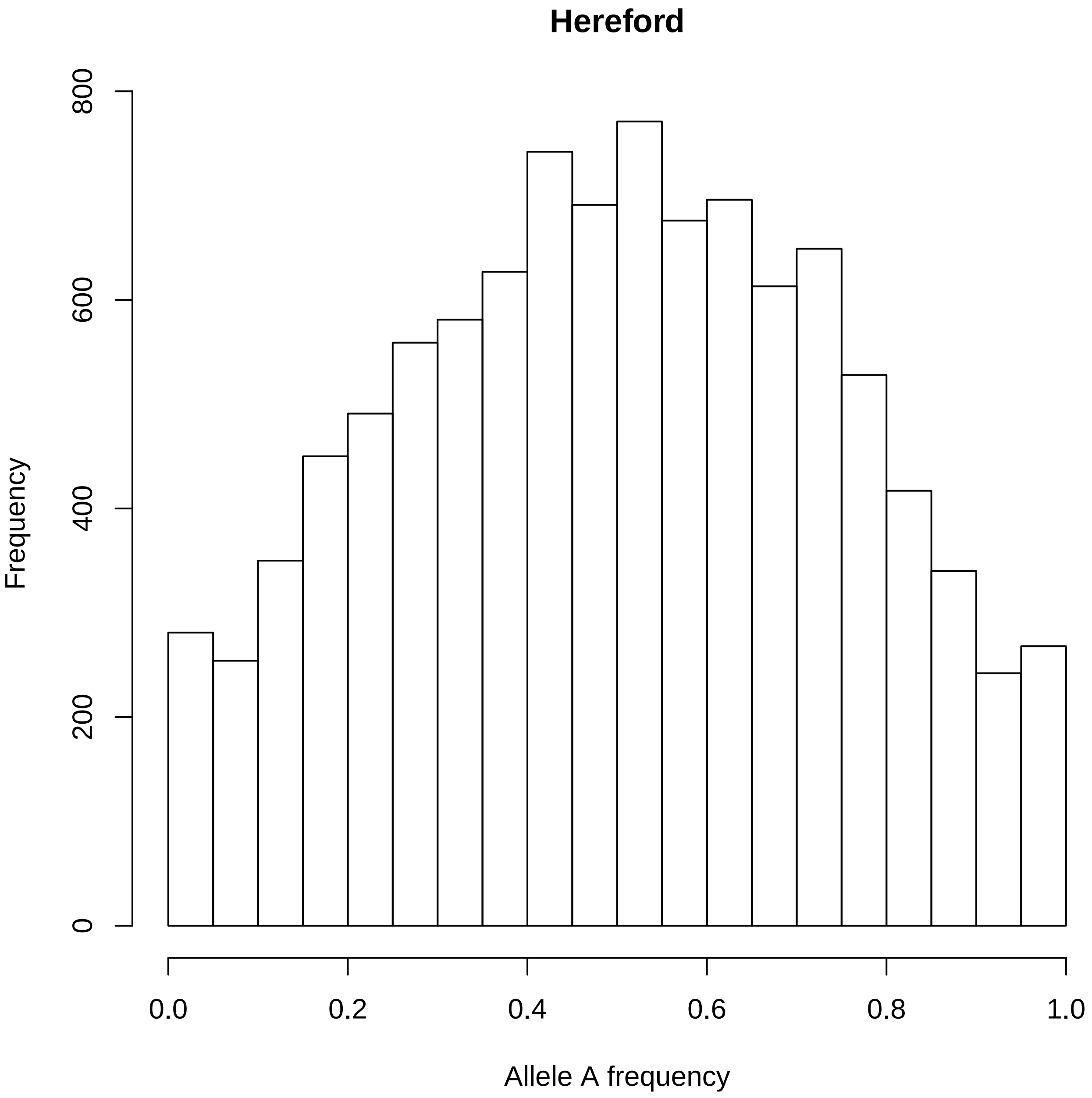
Hereford
Frequency
0
200
400
600
800
0.0
0.2
0.4
0.6
0.8
1.0
Allele A frequency

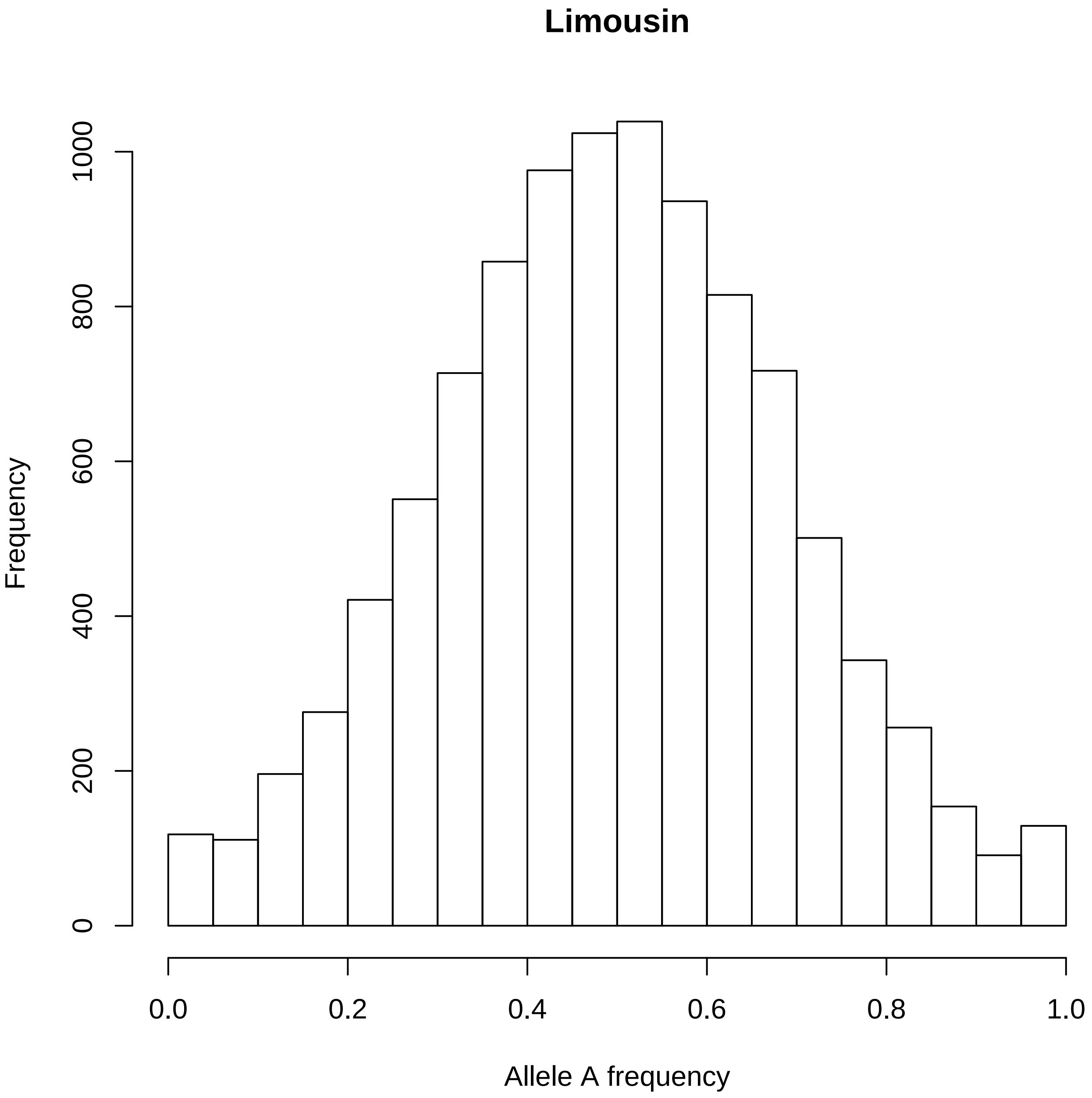

Limousin
Frequency
0
200
400
600
800
1000
0.0
0.2
0.4
0.6
0.8
1.0
Allele A frequency

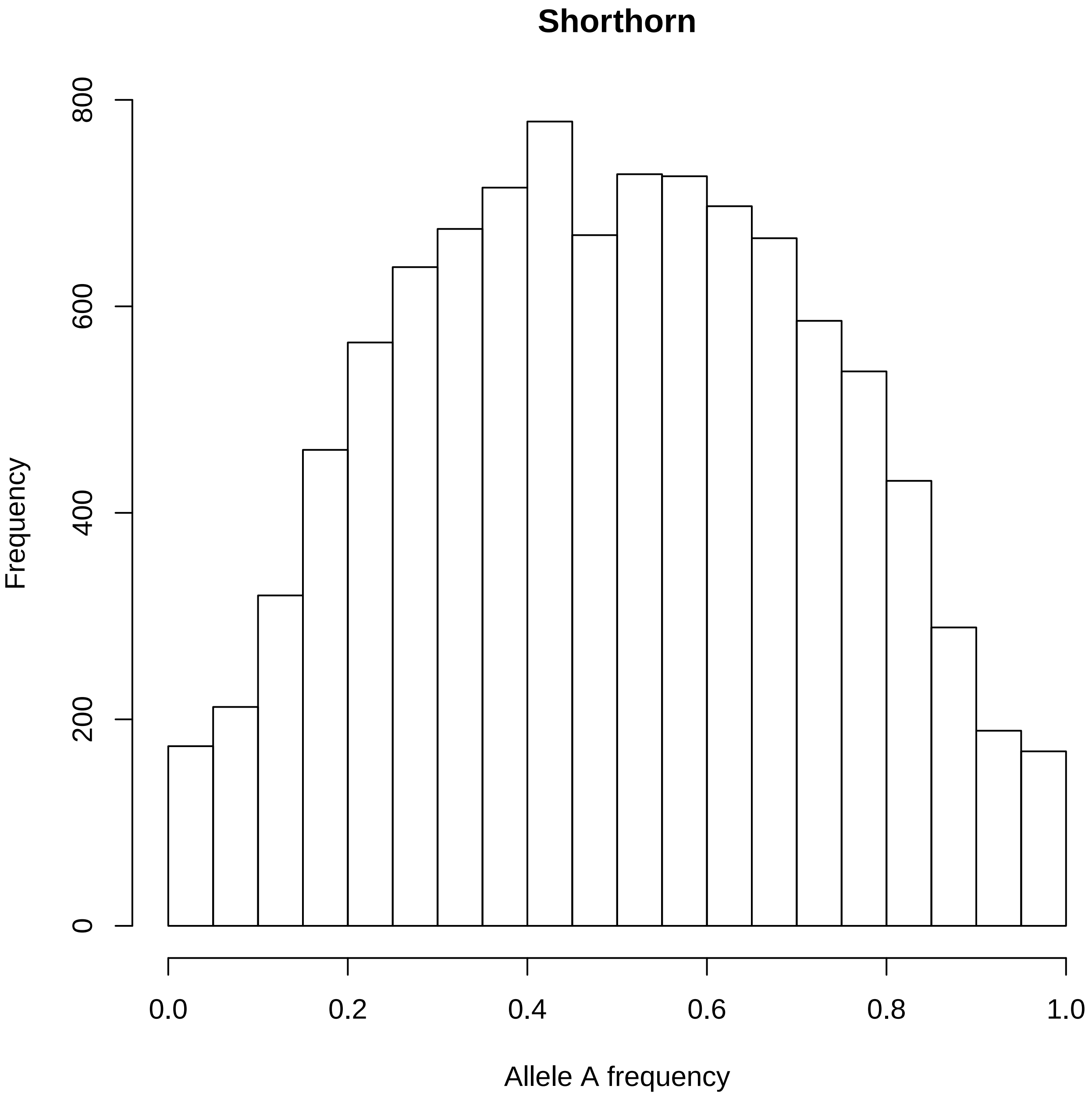

Shorthorn
Frequency
0
200
400
600
800
0.0
0.2
0.4
0.6
0.8
1.0
Allele A frequency

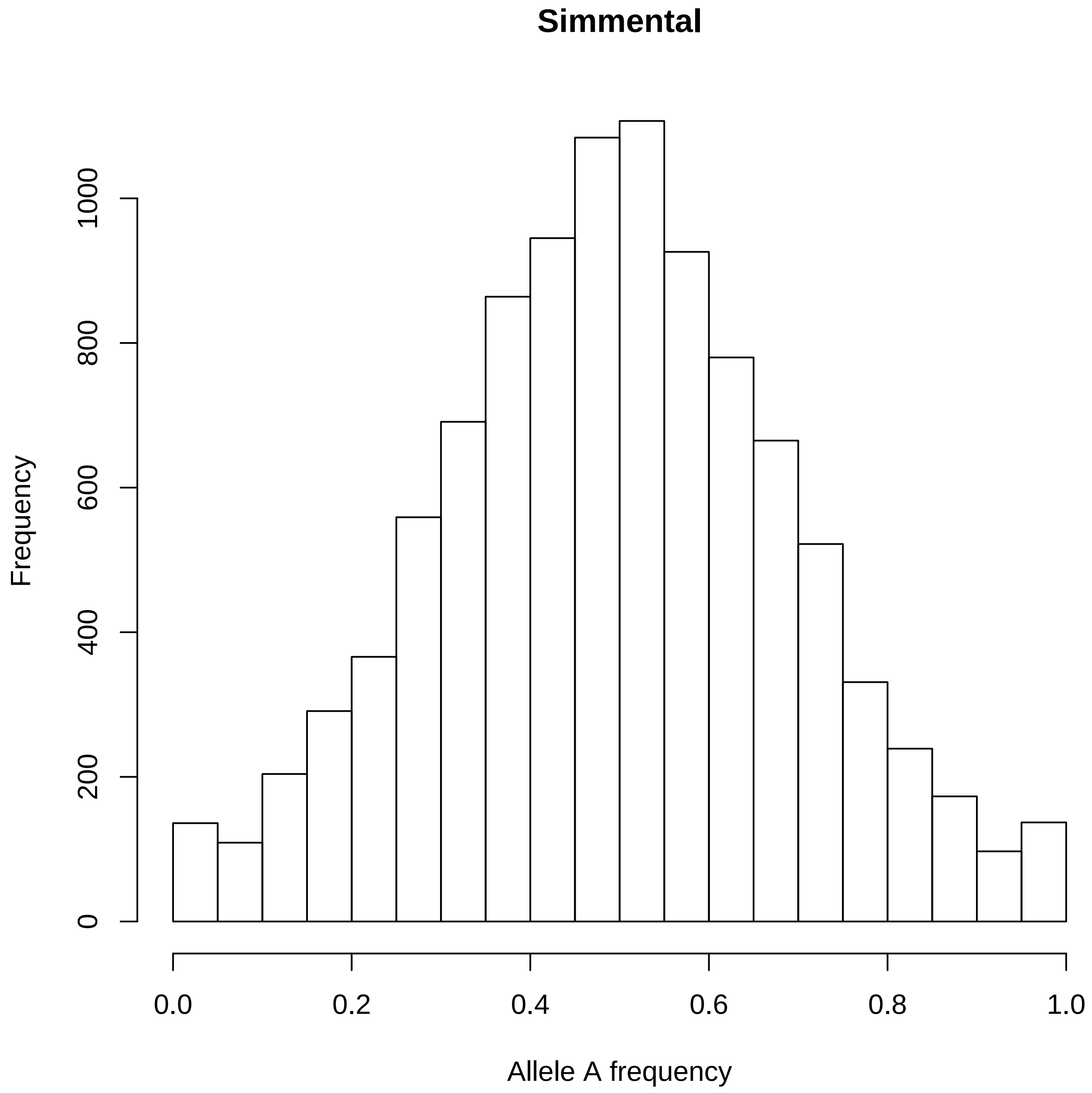
Simmental
Frequency
0
200
400
600
800
1000
0.0
0.2
0.4
0.6
0.8
1.0
Allele A frequency

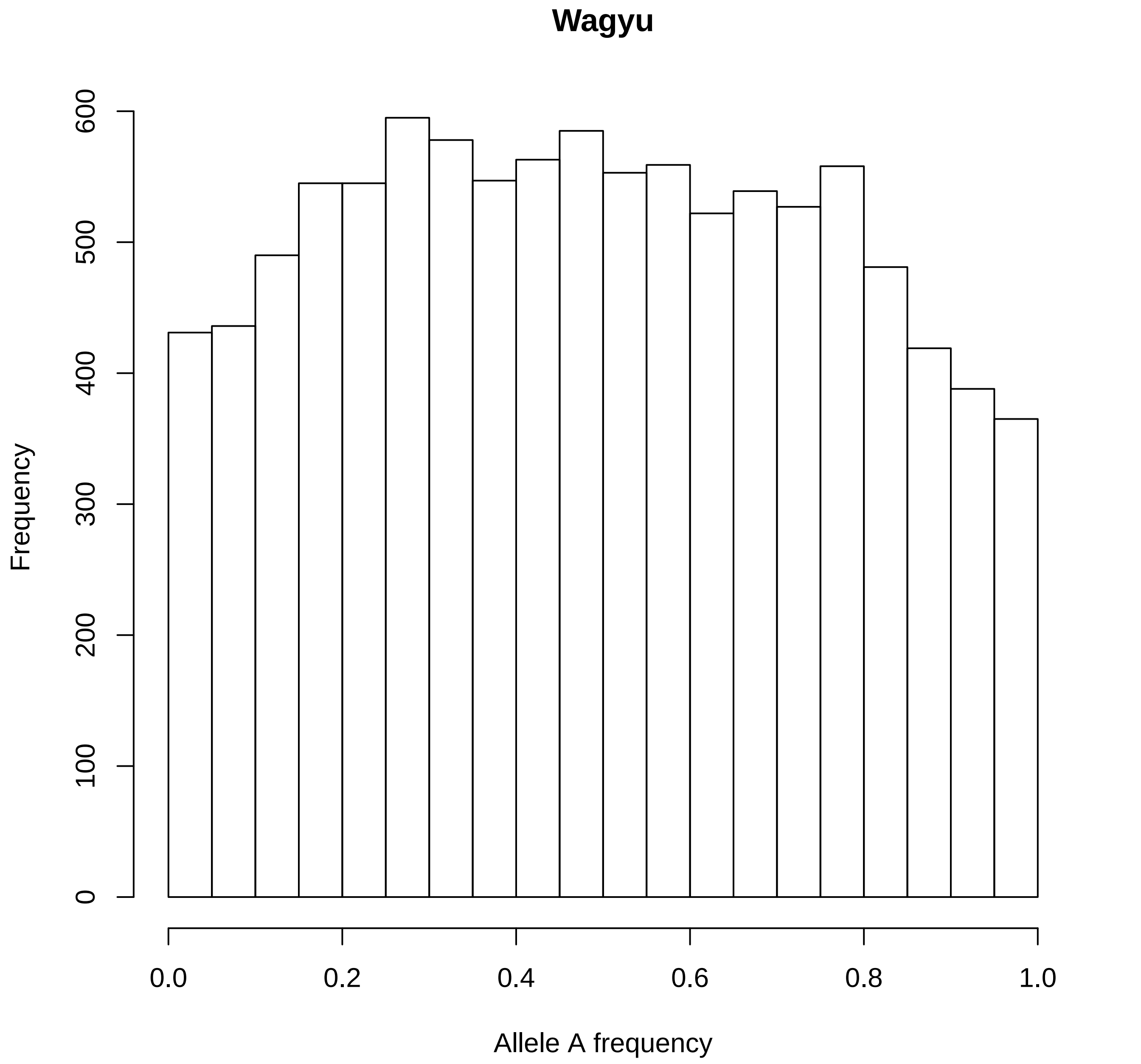
Wagyu
Frequency
0
100
200
300
400
500
600
0.0
0.2
0.4
0.6
0.8
1.0
Allele A frequency

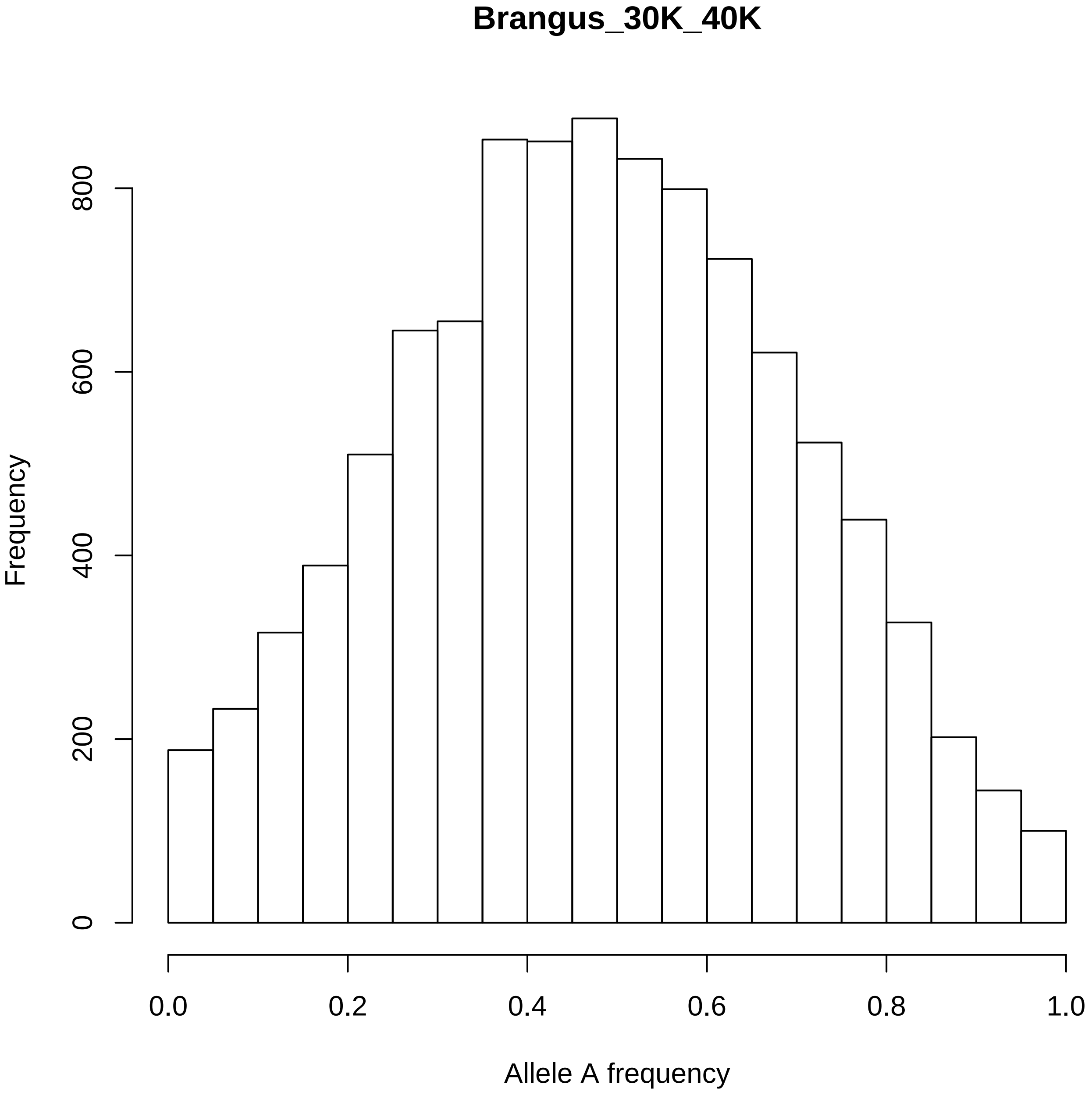
Brangus_30K_40K
Frequency
0
200
400
600
800
0.0
0.2
0.4
0.6
0.8
1.0
Allele A frequency

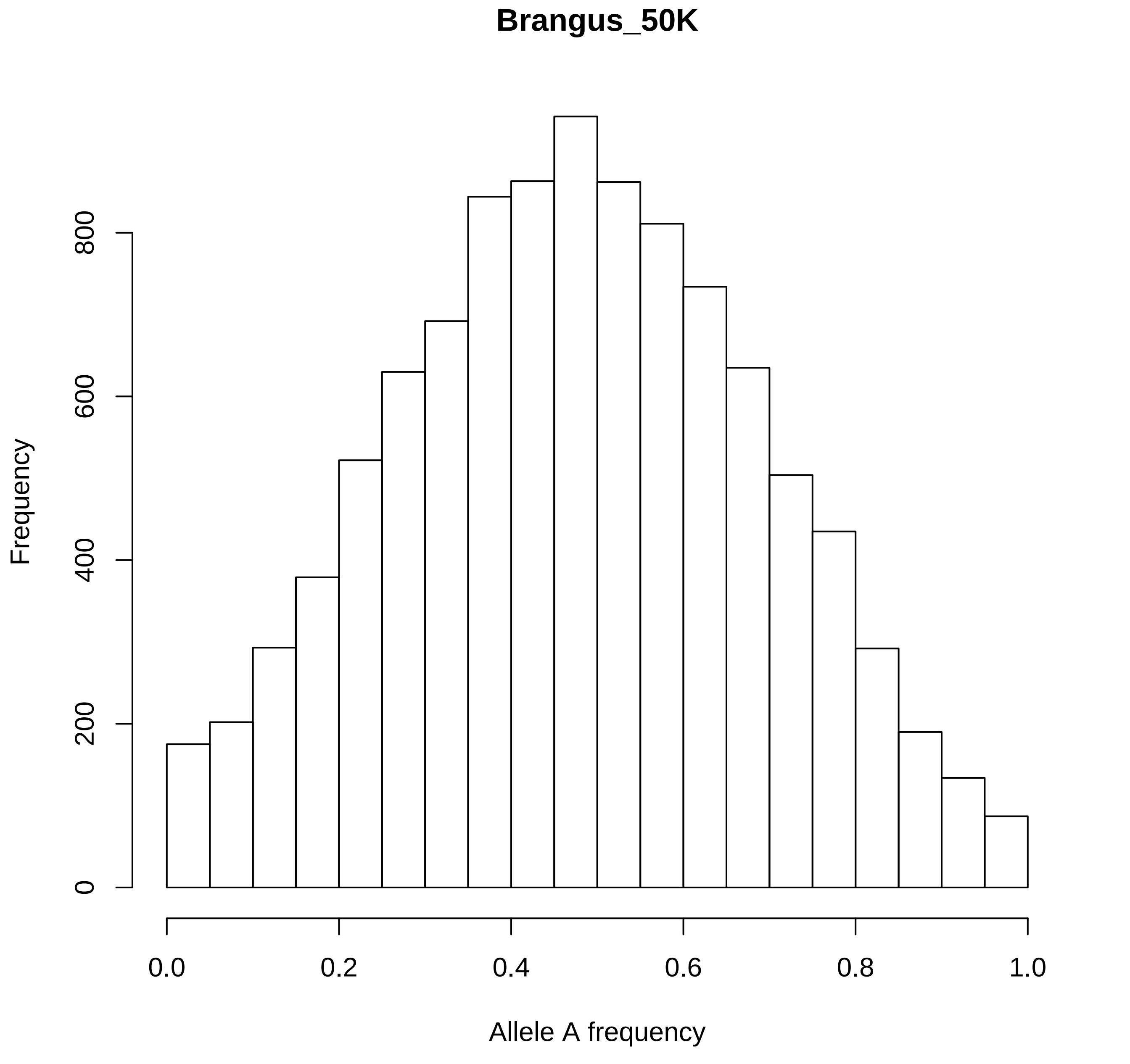

Brangus_50K
Frequency
0
200
400
600
800
0.0
0.2
0.4
0.6
0.8
1.0
Allele A frequency

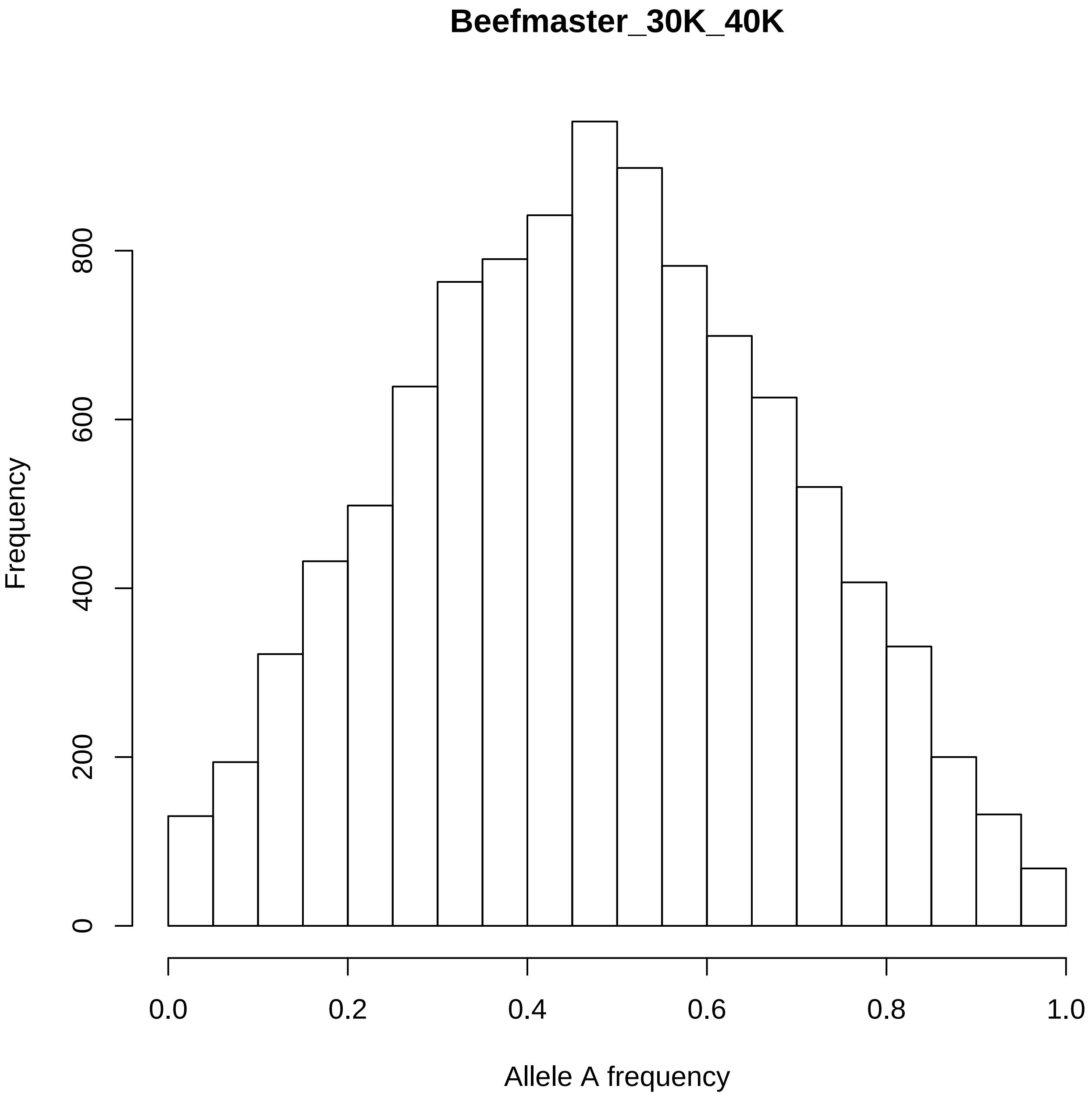
Beefmaster_30K_40K
Frequency
0
200
400
600
800
0.0
0.2
0.4
0.6
0.8
1.0
Allele A frequency

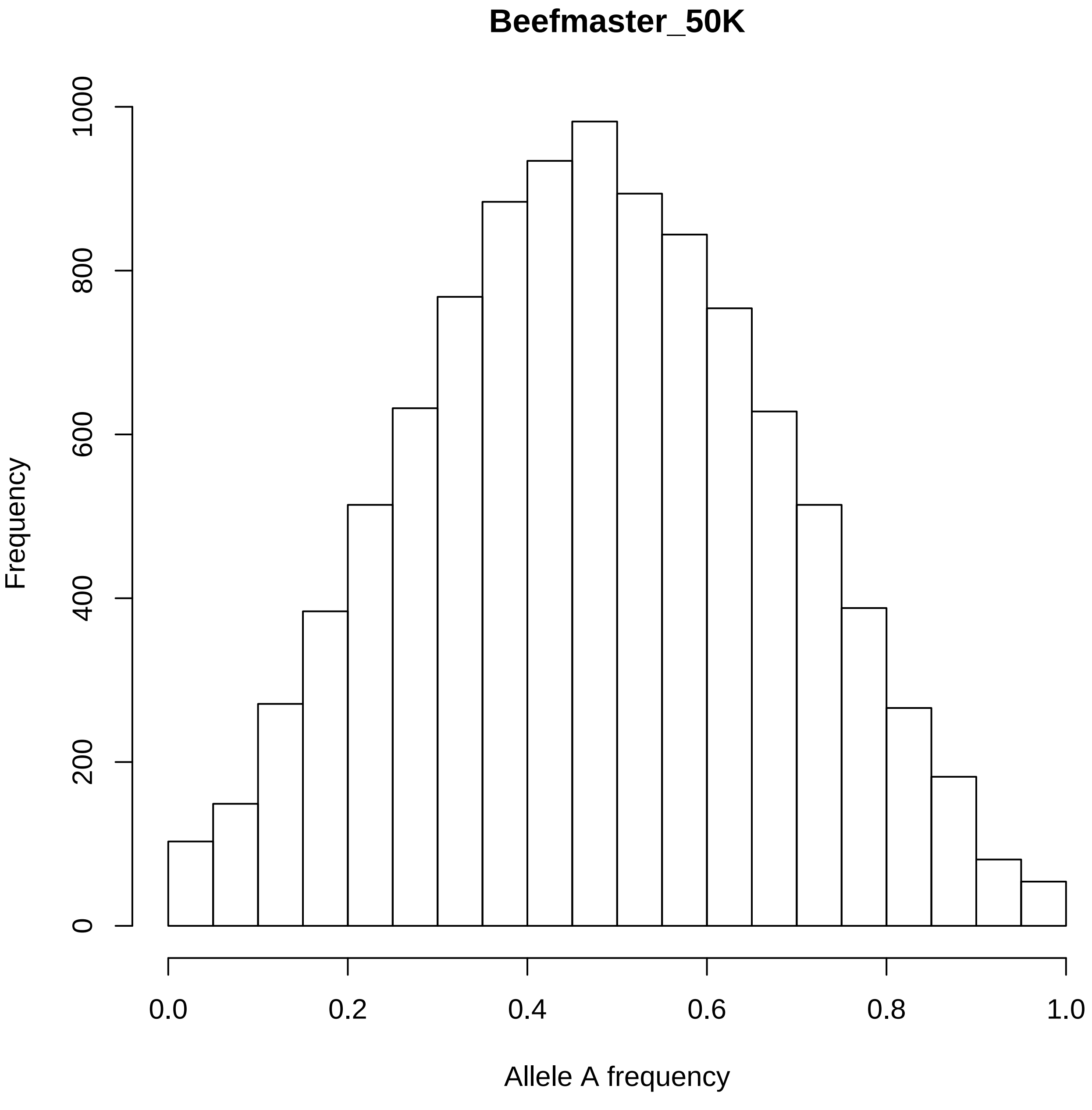
Beefmaster_50K
Frequency
0
200
400
600
800
1000
0.0
0.2
0.4
0.6
0.8
1.0
Allele A frequency
